# Correction to Efficient Targeted Degradation via Reversible and Irreversible Covalent PROTACs

**DOI:** 10.1021/jacs.0c05753

**Published:** 2020-06-10

**Authors:** Ronen Gabizon, Amit Shraga, Paul Gehrtz, Ella Livnah, Yamit Shorer, Neta Gurwicz, Liat Avram, Tamar Unger, Hila Aharoni, Shira Albeck, Alexander Brandis, Ziv Shulman, Ben-Zion Katz, Yair Herishanu, Nir London

This addition corrects several errors in the chemical drawings in the article. The correction has no influence on the data or conclusions of the work.

The configuration of the chiral carbon in Figure 1 in the main text was originally drawn as *S*. The corrected figure shown here depicts the *R* enantiomer used in this work.

**Figure 1:**
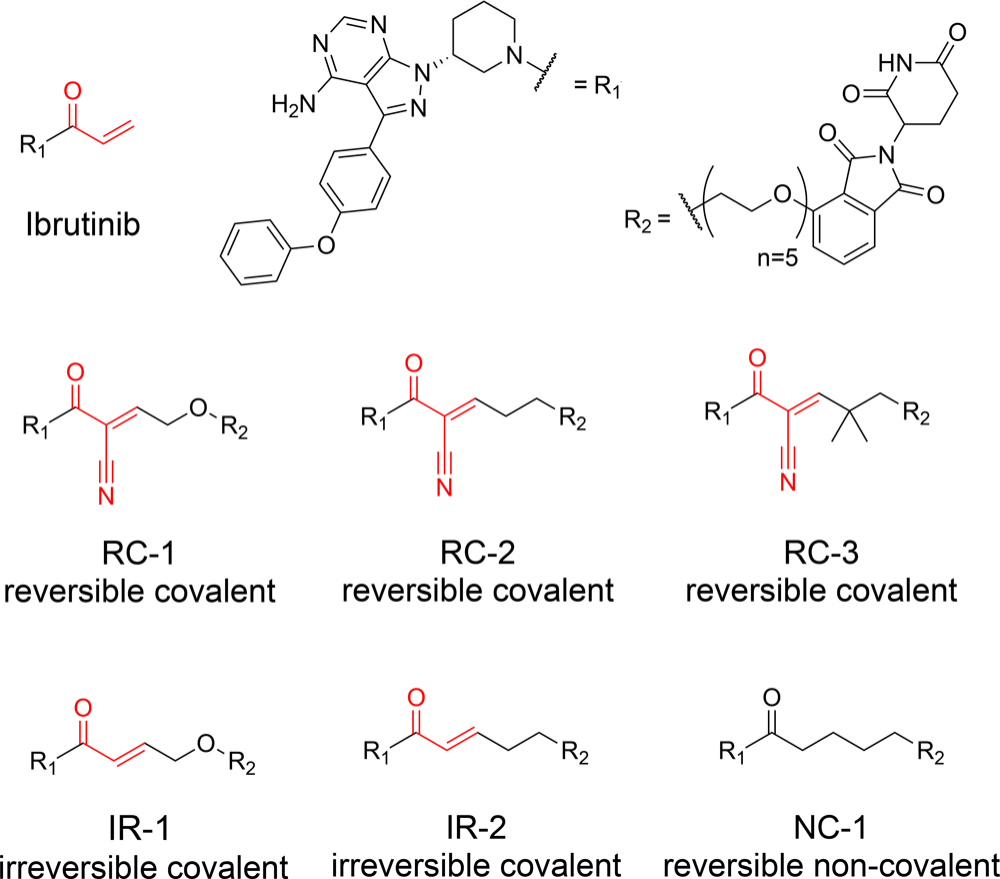
Structures of reversible covalent, irreversible covalent and noncovalent BTK PROTACs described in this study. The electrophilic moieties are highlighted in red.

In the Supporting Information PDF files, the configuration of the chiral carbon of the BTK binder in supplementary Table 1 (page S9), supplementary Figure 4 (page S13), and several of the synthetic schemes (pages S17–S42) was originally drawn and marked as *S* by error. The corrected supplementary file depicts the *R* enantiomer, which was the sole enantiomer used in the work.

The linker in the right panel of supplementary Table 1 (page S9) was missing two carbons in the drawing, and the linker size written for compound PG15 (RC-0b) in the table was incorrect. The corrected supplementary file contains the correct drawings and linker sizes.

## Supporting Information Available

Additional information including detailed experimental methods, description of the reversible covalent BTK PROTAC library and its degradation results in two cell lines, BTK degradation by PROTACs in Ramos cells, data on cellular penetration of PROTACs, time dependency of BTK degradation, validation of CRBN-mediated degradation, kinetic studies of BTK labeling using LC/MS, GSH effect on labeling, proteomics selectivity analysis for the PROTACs at additional concentrations, B cell receptor signaling inhibition data, detailed synthetic protocols for preparation of compounds with high-resolution mass spectrometry and NMR analysis (PDF)

