# Supplementary material for: Correction to Efficient Targeted Degradation via Reversible and Irreversible Covalent PROTACs
Source: J Am Chem Soc. 2020 Jun 10;142(25):11316. doi: 10.1021/jacs.0c05753 (PMC8506570; doi:10.1021/jacs.0c05753)
Supplement: Supplementary file 1 — ja0c05753_si_001.pdf [file ja0c05753_si_001.pdf]

Efficient targeted degradation via reversible and irreversible covalent PROTACs

Ronen Gabizon[1,*], Amit Shraga[1,*], Paul Gehrtz[1], Ella Livnah[1], Yamit Shorer[2], Neta Gurwicz[3], Liat Avram[4], Tamar Unger[5], Hila Aharoni[5], Shira Albeck[5], Alexander Brandis[6], Ziv Shulman[3], Ben-Zion Katz[7], Yair Herishanu[7], Nir London[1,#]

[1] Dept. of Organic Chemistry, The Weizmann Institute of Science, Rehovot, 7610001, Israel.

[2] Sackler Faculty of Medicine, Tel Aviv University, Tel-Aviv, Israel.

[3] Dept. of Immunology, The Weizmann Institute of Science, Rehovot, 7610001, Israel.

[4] Dept. of Chemical Research Support, The Weizmann Institute of Science, Rehovot 7610001, Israel.

[5] Structural Proteomics Unit, Department of Life Sciences Core Facilities, The Weizmann Institute of Science, Rehovot 7610001, Israel.

[6] Life Sciences Core Facilities, Weizmann Institute of Science, Rehovot 7610001, Israel

[7] Department of Hematology, Tel Aviv Sourasky Medical Center, Tel Aviv, Israel and Sackler Faculty of Medicine, Tel Aviv University, Tel-Aviv, Israel.

[*] equal contribution

[#] To whom correspondence should be addressed, nir.london@weizmann.ac.il

Keywords: PROTACs, reversible covalent, cyanoacrylamides, BTK, targeted degradation

Table of Contents for Supplementary Material

Supplementary methods…………………………………………………………………S3
Supplementary tables and figures………………………………………………………...S9
Chemistry Methods………………………………………………………………………S17

Supplementary Methods

Cloning and Over Expression of BTK

Human wild type BTK full length was obtained from the ORFeome (ref: http://www.orfeomecollaboration.org/), from which the full human BTK gene (clone HsCD00433457) was inserted into the mammalian expression vector pCDNA3.1. In parallel, a mutant BTK C481S was constructed by assemble 2 PCR products harboring the desired mutation and cloned into pCDNA3.1. Both constructs contain a C-terminal 6x-His tag and three amino acids sequence (GTK) linker between the BTK coding sequence and the His tag. The expression of hBTK is driven by the CMV promoter. U2OS cells (ATCC HTB-96) were grown to 70% confluency in a 10 cm plate, transfected with either hBTK plasmid (BTK WT or C481S) using Lipofectamine 2000 reagent (Thermo Fisher Scientific) according to manufacturer's instructions. Cells were subsequently incubated for 48 hours post transfection, trypsinized and resuspended in 12 mL medium, and divided 2 mL per well in a 6-well plates and left to adhere over-night. 72 hours post transfection 100 nM PROTACs were added for 24 hours and BTK was measured by performing western blot procedure as described in the main text.

Expression and Purification of Recombinant BTK

The expression and purification of human BTK kinase domain (residues 387–659) was based on the method used by Bradshaw JM. *et al*[1]. The kinase domain was inserted into pFastBac-1 with an N-terminal 6×-His tag followed by a TEV protease cleavage site (The plasmid was a gift from Dr. Ville Paavilainen, University of Helsinki). Viruses were produced in Sf9 cells and expression of the BTK kinase domain was subsequently induced in Tni insect cells by infection of 2 L of cultured cells with 1:200 mL virus solution such that cell growth was terminated after 3 days. The cells were collected by centrifugation (800 g for 15 minutes), and the pellet was resuspended in 50 mL lysis buffer (10 mM Hepes, pH 7.5, 400 mM NaCl, 1.5 mM DTT) supplemented with 1× protease inhibitor cocktail (Roche). The cells were lysed by five passages through a cell homogenizer. The cellular debris were pelleted by centrifugation (30,000 g for 30 minutes). The protein was bound in batch to nickel–nitrilotriacetic acid agarose beads in binding buffer (lysis buffer supplemented with 20 mM imidazole) for 4 hours at 4°C. The beads were washed with additional binding buffer (four 5 mL washes), and the protein was eluted with four 0.5 mL portions of elution buffer (lysis buffer

supplemented with 300 mM imidazole). The His tag was cleaved by the addition of TEV protease with concomitant dialysis overnight into cleavage buffer (50 mM Tris, pH 8.0, 0.5 mM EDTA, 1 mM DTT) at 4°C. The resulting soluble protein was passed over Ni-NTA beads to remove the protease and purified further by gel filtration on a HiLoad_16/60_Superdex_75 (GE Healthcare) column equilibrated with 20 mM Tris, pH 8.0, 50 mM NaCl, 1 mM DTT. The pure protein was then flash-frozen in liquid nitrogen and stored at −80°C.

B Cell Activation

C57BL/6 mice were purchased from Harlan (Rehovot, Israel) and maintained in a pathogen-free facility, experiments were carried on 8-12 weeks old male mice. All experiments with mice were approved by the Weizmann Institute Animal Care and Use Committee.

Splenic cells from C57BL/6 mice were isolated by forcing spleen tissue through mesh into PBS containing 2% fetal calf serum and 1 mM EDTA and red blood cells were depleted by lysis buffer. Cells were cultured in 96-well U-bottom dishes ($1x10^6$ cells/mL in RPMI 10% FCS) and incubated with BTK PROTACs or Ibrutinib in different concentrations (1 µM, 100 nM, 10 nM, 1 nM, 0.1 nM) for 24 hours at 37°C in 5% humidified CO_2 incubator. Following a 24 hours incubation, cells were stimulated with anti-IgM overnight (5 µg/mL, Sigma-Aldrich). Subsequently, cells were stained with anti-B220 (clone RA3-6B2, Biolegend) and anti-CD86 (clone GL-1, Biolegend) antibodies for 30 minutes at 4°C. Single cell suspensions were analyzed by a flow cytometer (CytoFlex, Beckman Coulter).

Proteomics

Cells were analyzed by intensity based, label-free mass spectrometry based proteomics. Sample preparation: 10^6 Ramos cells were treated in four replicates with either compound or DMSO for 24 hours. Cells were then washed twice by centrifuge at 200 rcf, 4°C for 5 minutes, removing the supernatant and washed with ice cold PBS. Samples were then centrifuged at 200 rcf, 4°C for 5 minutes, then supernatant was removed and samples were frozen at -80°C. Samples were dispersed in 75 µl of 50 mM ammonium bicarbonate, and transferred to 1.8 mL glass vials. 75 µl of 10% SDS in 50 mM ammonium bicarbonate were added and the samples were heated to 96°C for 6 minutes. The samples were sonicated thoroughly in a sonication bath until the DNA was sheared as indicated by reduction in viscosity to level enabling easy pipettation. Total protein concentration was estimated using BCA assay and 30 µg from each sample was taken for the experiment.

The samples were reduced by the addition of 1/20 volume of 100 mM DTT and heating to 60°C for 45 minutes. The samples were cooled to room temperature, and 1/20 volume of 200 mM iodoacetamide was added and the reaction was performed in the dark for 30 minutes.

At this point, 1/10 volume of 12% phosphoric acid was added, followed by 6 volume of 90% methanol in 50 mM ammonium bicarbonate. The samples were loaded on S-trap Micro columns (Protify), and washed 3 times with 90% methanol in 50 mM ammonium bicarbonate. The columns were spun without washing to dry the methanol. Then, 1 μg of trypsin (Promga) in 20 μl of 50 mM ammonium bicarbonate was added to each column, and they were incubated at 47°C for 1.5 hours. Then, 40 μl of 50 mM ammonium bicarbonate was added and the sample was eluted and another 0.5 μg of trypsin was added, followed by overnight incubation at 37°C. The columns themselves were further eluted with 40 μl 0.2% formic acid in water, followed by 40 μl of 0.2% formic acid in 50% acetonitrile:water, to elute strongly bound peptides. This elution was stored at 4°C and combined the next day with the first elution. The samples were then dried by SpeedVac and analyzed.

Liquid chromatography: ULC/MS grade solvents were used for all chromatographic steps. Each sample was loaded using split-less nano-Ultra Performance Liquid Chromatography (10 kpsi nanoAcquity; Waters, Milford, MA, USA). The mobile phase was: A) H2O + 0.1% formic acid and B) acetonitrile + 0.1% formic acid. Desalting of the samples was performed online using a reversed-phase Symmetry C18 trapping column (180 µm internal diameter, 20 mm length, 5 µm particle size; Waters). The peptides were then separated using a T3 HSS nano-column (75 µm internal diameter, 250 mm length, 1.8 µm particle size; Waters) at 0.35 µL/minute. Peptides were eluted from the column into the mass spectrometer using the following gradient: 4% to 30% B in 150 minutes, 35% to 90% B in 5 minutes, maintained at 90% for 5 minutes and then back to initial conditions.

Mass Spectrometry: The nanoUPLC was coupled online through a nanoESI emitter (10 μm tip; New Objective; Woburn, MA, USA) to a tribrid Orbitrap Fusion Lumos mass spectrometer (Thermo Scientific) using a PicoView nanospray apparatus (New Objective). Data was acquired in data dependent acquisition (DDA) mode, using a top-speed method with maximum cycle time of 3 sec. MS1 resolution was set to 120,000 (at 200 m/z), mass range of 375-1650 m/z, AGC of 4e5 and maximum injection time was set to 50 msec. MS2 was performed by HCD in the Orbitrap with resolution set to 15,000, quadrupole isolation 1 Th, AGC of 5e4, and maximum injection time of 100 msec.

Raw data were analyzed using the MaxQuant software suite 1.6.0.16 (www.maxquant.org) with the Andromeda search engine[2]. The higher-energy collisional dissociation (HCD) MS/MS spectra were searched against an in silico tryptic digest of *Homo sapiens* proteins from the UniProt/Swiss-Prot sequence database (v. July 2019), including common contaminant proteins. All MS/MS spectra were searched with the following MaxQuant parameters: acetyl (protein N-terminus) and methionine oxidation as variable modifications; cysteine carbamidomethylation was set as fixed modification for all samples, except for the SDT samples in which case carbamidomethylation was set as variable modification; max 2 missed cleavages; and precursors were initially matched to 4.5 ppm tolerance and 20 ppm for fragment spectra. Peptide spectrum matches and proteins were automatically filtered to a 1% false discovery rate based on Andromeda score, peptide length, and individual peptide mass errors. Processing was conducted without a match between runs.

Proteins were identified and quantified based on at least two unique peptides and based on the label-free quantification (LFQ)[3] values reported by MaxQuant. Every data set contained three sets of four replicates (one set for DMSO treated samples, one set for 50 nM and one set for 100 nM). Proteins were excluded from analysis if within all three sets, there were less than three samples with directly detected and quantified protein. After this filtering step, missing values were replaced from a normal distribution.

Metabolomics

Sample preparation: 10^6 Ramos cells were treated in three replicates with 100 nM compound for 2 hours. Cells were then washed twice by centrifuge at 200 rcf, 4°C for 5 minutes, removing the supernatant and washed with ice cold PBS. Samples were then centrifuged at 200 rcf, 4°C for 5 minutes, then supernatant was removed and samples were frozen at -80°C. Samples were dispersed in 100 µl of PBS. From each sample, 20 µl were mixed with 20 µl of 10% SDS in 50 mM ammonium bicarbonate, heated to 96°C for 5 minutes, and the protein concentration was measured by BCA assay. Another 75 µl were mixed with 0.5 mL of 50% acetonitrile:water, sonicated 3 times 10 seconds on ice, centrifuged for 10 minutes at 15000 rpm at 4°C, and the supernatant was transferred to a new tube and lyophilized. The residue was dissolved in 25% acetonitrile in water in a volume calculated from the BCA assay to obtain a concentration equivalent to 1.09 µg / µl protein. The samples were centrifuged at 21,000 g for 5 minutes to remove insoluble material. Standard curves from 0.1-100 ng/mL for each compound were used for quantitation.

The LC–MS/MS instrument consisted of an Acquity I-class UPLC system (Waters) and Xevo TQ-XS triple quadrupole mass spectrometer (Waters) equipped with an electrospray ion source. Chromatographic separation was done on UPLC BEH C18 column (50 mm × 2.1 mm, 1.7-µm, Waters) using 50-100% gradient of 95%-aqueous acetonitrile with 0.1% formic acid in aqueous 0.1% formic acid during 2.5 minutes, with a flow rate of 0.3 mL min^{-1} and column temperature 30°C. Samples kept at 10°C were automatically injected in a volume of 3 µl.

MS parameters (positive ion mode): capillary voltage - 2.0kV, source temperature - 150°C. MRM transitions, m/z (collision energy, eV): 970.3 > 84.0 (62), 970.3 > 301.0 (47) and 970.3 > 304.0 (57) for RC-2; 945.2 > 84.0 (75), 945.2 > 301.0 (50) and 945.2 > 304.2 (68) for IR-2; 947.2 > 84.0 (70), 947.2 > 304.1 (72) and 947.2 > 387.0 (50) for NC-1; and 998.2 > 84.0 (75), 998.2 > 301.0 (55) and 998.2 > 304.1 (70) for RC-3.

Targeting BTK in CLL cells

Patients and samples

After signing an informed consent form approved by the Tel Aviv Medical Center IRB according to the Helsinki Accords, blood samples were collected from patients fulfilling the standard criteria for CLL. Peripheral blood mononuclear cells (PBMC) were isolated by Ficoll (Uppsala, Sweden) density-gradient centrifugation. Viable frozen cells were kept in FCS (Biological Industries, Beit-Haemek, Israel) containing 10% DMSO (Merck, Darmstadt, Germany) and stored in liquid nitrogen. Before use, frozen cells were thawed and cultured at 37°C, 5% CO_2, in RPMI medium supplemented with 10% FCS, penicillin, streptomycin, and L-glutamine (all from Biological Industries, Beit-Haemek, Israel.)

Antibodies and Reagents

Anti-BTK antibody was purchased from Cell Signaling Technology (Beverly, MA) and anti-actin form MP Biomedicals (Illkirch, France). Goat anti Rabbit IgG (H+L)-HRP conjugate and Goat anti Mouse IgG (H+L)-HRP conjugate were from Jackson ImmunoResearch laboratories (West Grove, PA). All antibodies utilized in the study were used in concentrations according to the manufacturer's instructions.

Degradation Experiments and Western Blotting

CLL cells ($20x10^6$/mL) were incubated with PROTACs, at the indicated doses, at 37°C for 18 hours. The PROTACs were dissolved in DMSO, and controls were treated with DMSO accordingly.

Following incubation with PROTACs as detailed, cells were collected and lysed in cell lysis buffer (Cell Signaling Technology, Beverly, MA) containing Phosphatase Inhibitor Cocktail 2 and protease inhibitor Cocktail (Sigma-Aldrich, St. Louis, MO). Extract from cell lysates were separated on 4–15% Criterion™ TGX™ Precast Midi Protein Gel (BioRad), and transferred electrophoretically to nitrocellulose paper. The nitrocellulose paper was incubated with the designated antibodies and HRP conjugated secondary antibodies according to the manufacturer's instructions. Bands were detected using MyECL Imager (Thermo Scientific, Rockford, IL).

Supplementary Tables and Figures:

Supplementary Table 1: Reversible covalent BTK PROTAC library

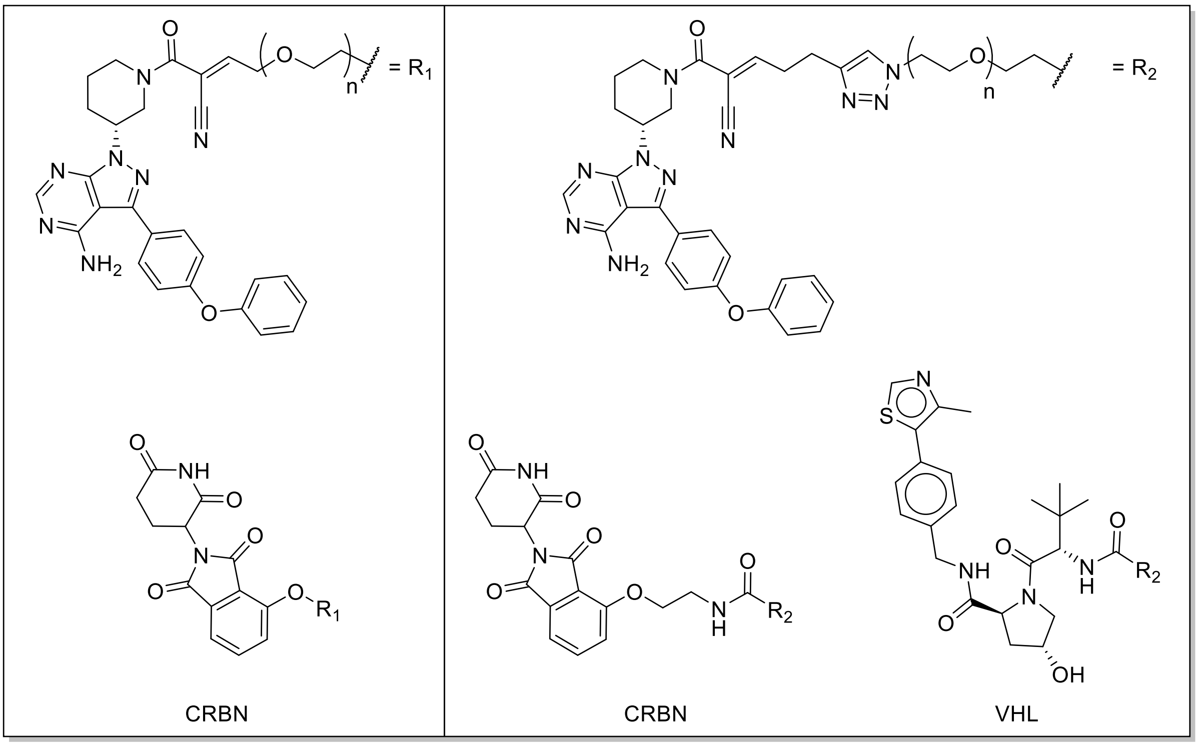

Direct PEG linkage

Triazole Linkage

**Compound**	**E3 binder**	**Linker size (n=)**	**Linker Chemistry**
PG37 (RC-0a)	R_1 = CRBN	4	Direct PEG
**PG27** (RC **-1)**	R_1 = CRBN	5	Direct PEG
PG15 (RC -0b)	R_1 = CRBN	2	Direct PEG
RG16 (RC -0c)	R_2 = VHL	6	Triazole
RG15 (RC -0d)	R_2 = VHL	4	Triazole
RG14 (RC -0e)	R_2 = VHL	3	Triazole
RG13 (RC -0f)	R_2 = VHL	2	Triazole
RG12 (RC -0g)	R_2 = CRBN	4	Triazole
RG11 (RC -0h)	R_2 = CRBN	3	Triazole
RG10 (RC -0i)	R_2 = CRBN	2	Triazole
RG8 (RC -0j)	R_2 = CRBN	6	Triazole

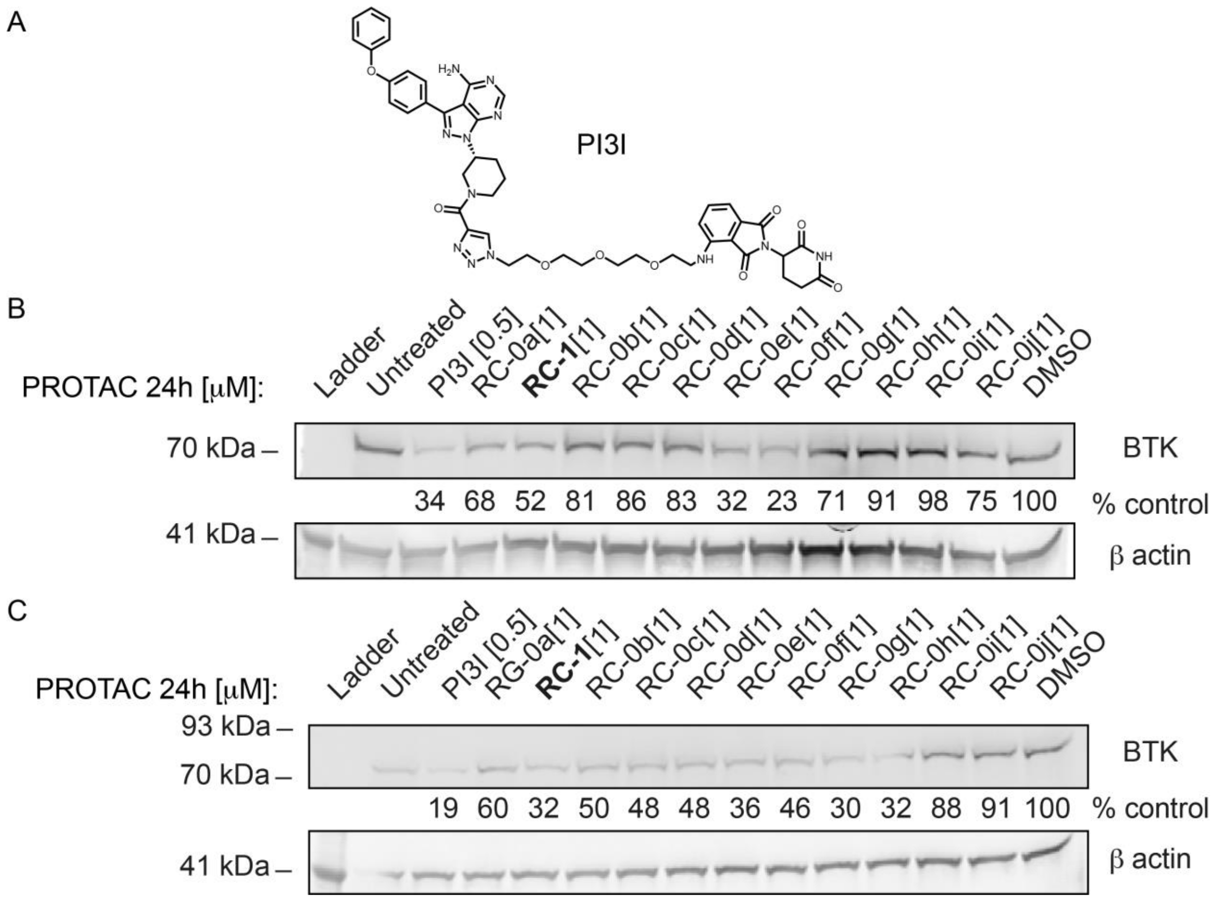

Supplementary Figure 1: Library screen for BTK PROTACs highlights RC-1 as a prominent PROTAC.

A. Chemical structure of PI3I, a previously reported non-covalent BTK PROTAC[4].

B. Western blot of Mino cells treated with the reversible covalent library of BTK PROTACs.

C. Western blot of K562 cells treated with the reversible covalent library of BTK PROTACs.

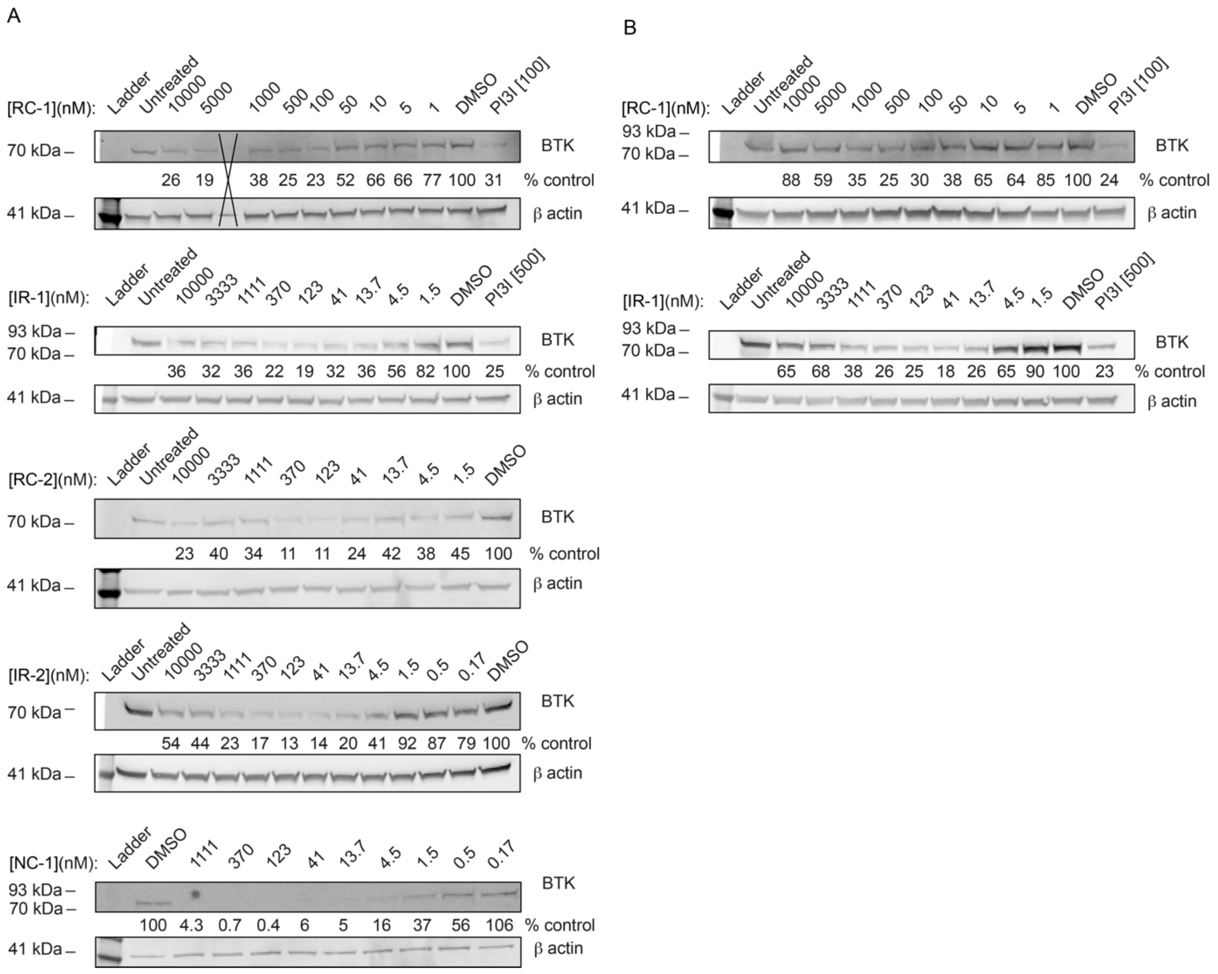

Supplementary Figure 2: BTK degradation by PROTACs

A. Western blot depicting a full dose-response curve for PROTACs in Ramos cell lines.

B. Western blot depicting a full dose-response curve for PROTACs in Mino cell lines.

Both experiments are after 24h incubations with the PROTACs.

Supplementary Table 2: Metabolomic quantification of PROTACs cellular concentration

PROTAC	Effective cellular concentration (pg compound / µg total protein)
NC-1	8.6 ± 2.6
IR-2	4.1 ± 0.7
RC-2	0.93 ± 0.06
RC-3	10.7 ± 3.1

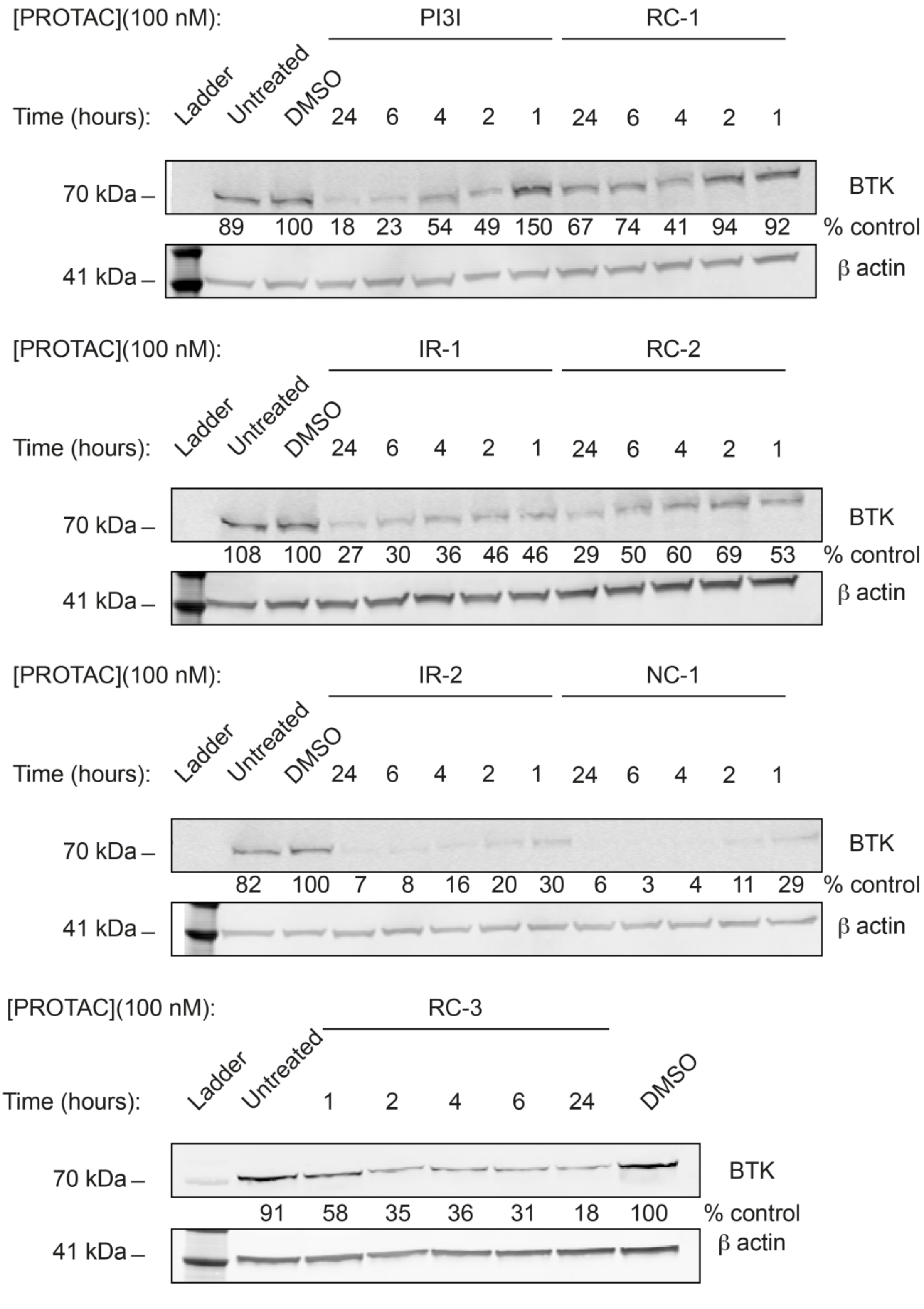

Supplementary Figure 3: BTK degradation time dependency.

Ramos (**PI3I, RC-1, IR-1, RC-2, IR-2, NC-1**) or Mino (**RC-3**) cells were treated with 100 nM of PROTAC or DMSO for indicated times, subsequently harvested for BTK levels measurement via western blot.

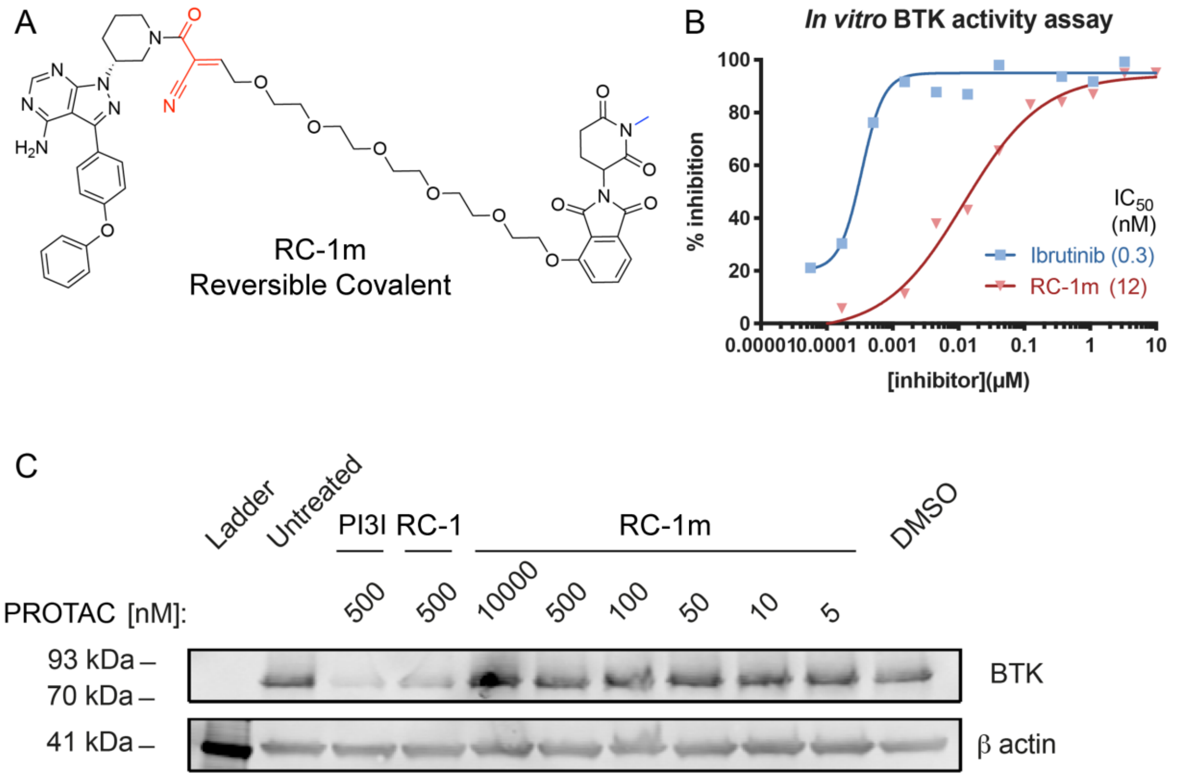

Supplementary Figure 4: Reversible covalent PROTAC induced degradation is mediated by CRBN.

A. Structure of **RC-1m**, an analog of **RC-1**, with a methylated (blue) CRBN binder, that abrogates CRBN binding.

B. *In vitro* kinase assay in the presence of Ibrutinib or **RC-1m**, shows that BTK binding is not hampered by this methylation (compare to **RC-1** in Fig. 2A)

C. Western blot depicting a full dose-response curve for **RC-1m** in Mino cell lines, shows no apparent degradation.

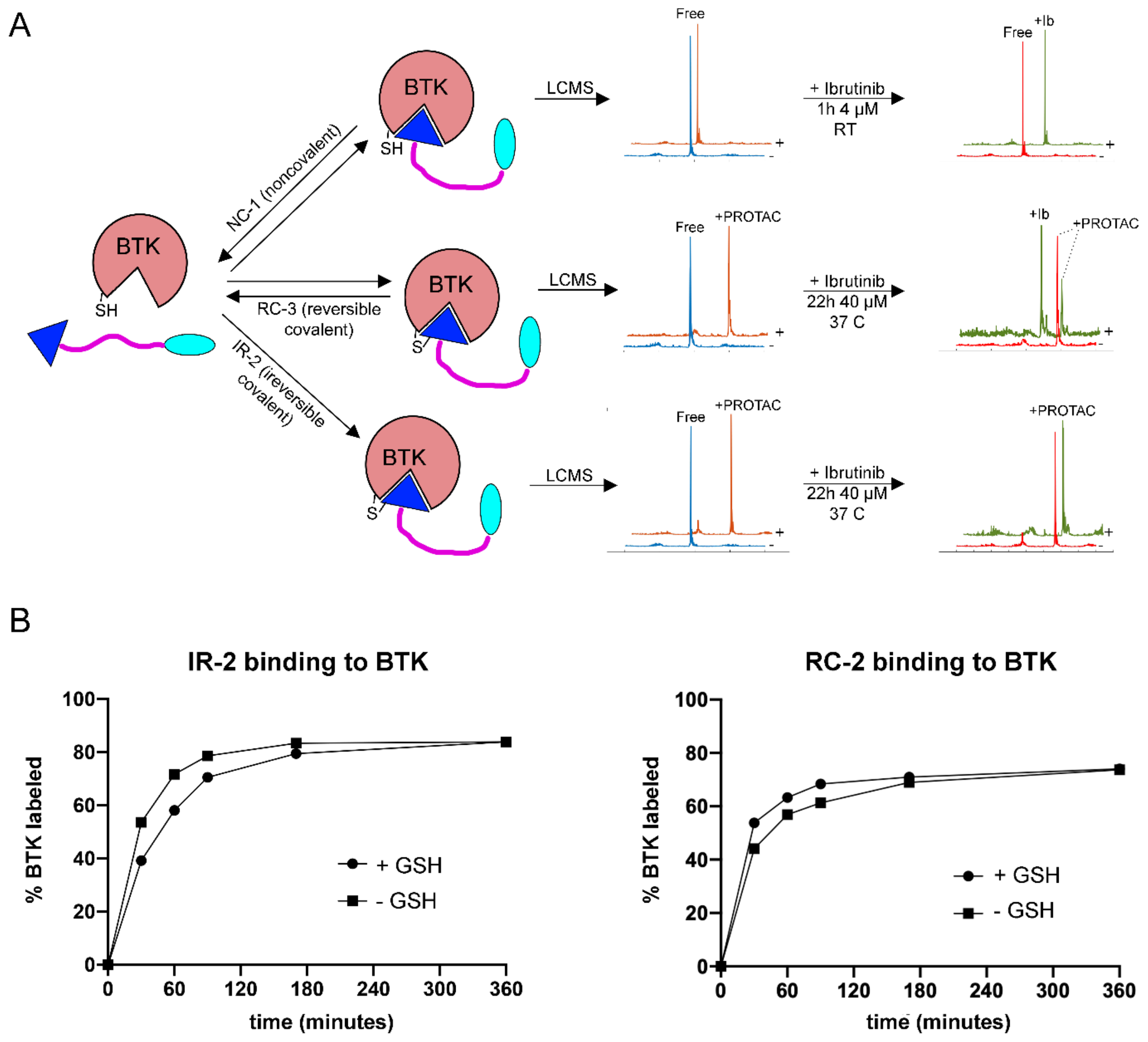

Supplementary Figure 5: Kinetic studies of BTK labeling using LC/MS

A. BTK was incubated with 3 μM compound for 2 hours at 25°C, followed by addition of Ibrutinib and incubation at indicated times and temperatures. Samples were analyzed before and after addition of Ibrutinib by intact LC/MS at indicated times.

B. Binding experiments in the presence of glutathione. Compounds (4 μM) were incubated with glutathione (6.14 mM) in assay buffer for 30 minutes, followed by addition of BTK to 2 μM (which diluted the compounds to 3.25 μM and the glutathione to 5 mM) and monitoring of the binding reaction by LC/MS.

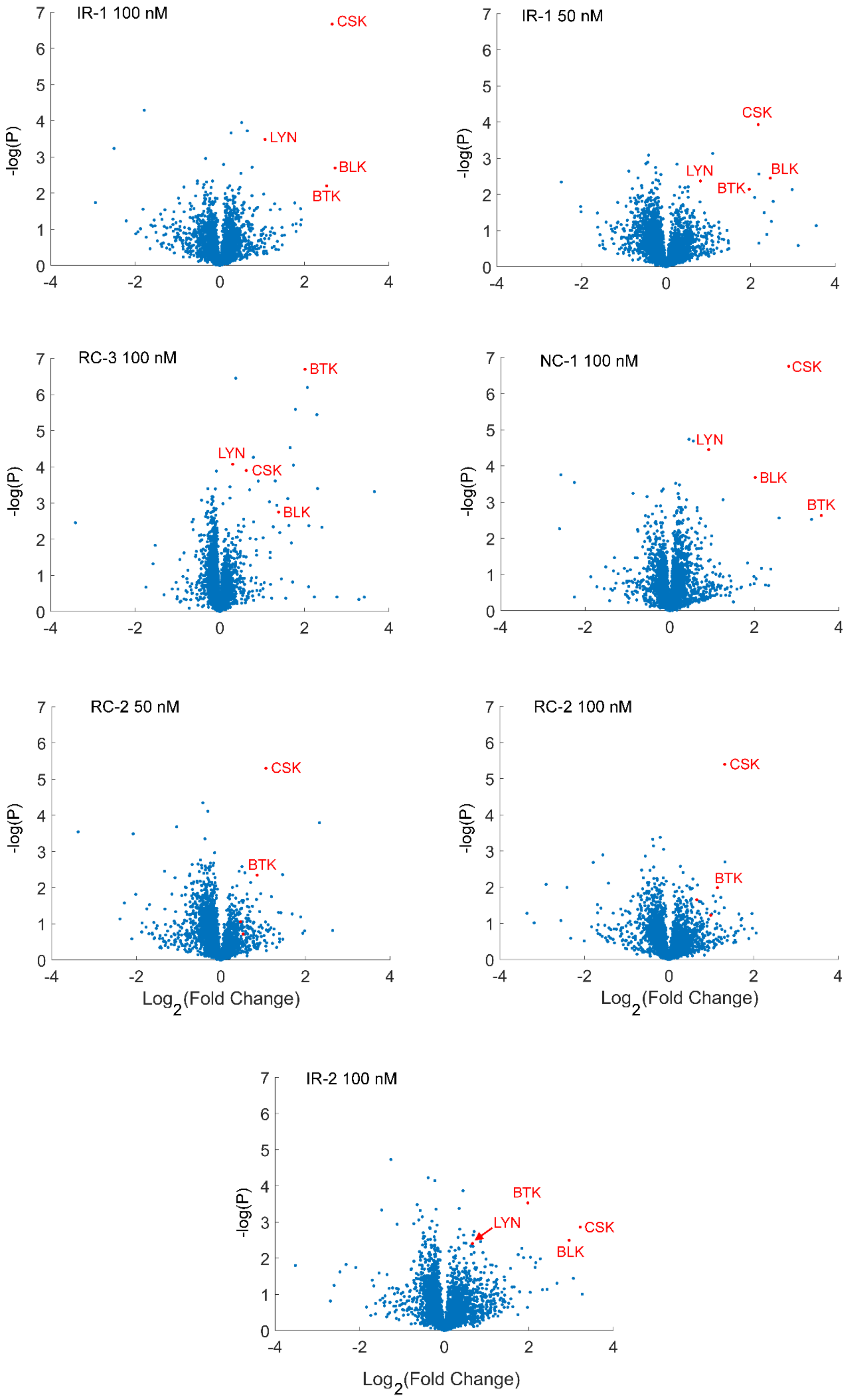
IR-1 100 nM
CSK
LYN
BLK
BTK
IR-1 50 nM
CSK
LYN
BLK
BTK
RC-3 100 nM
BTK
LYN
CSK
BLK
NC-1 100 nM
CSK
LYN
BLK
BTK
RC-2 50 nM
CSK
BTK
RC-2 100 nM
CSK
BTK
IR-2 100 nM
BTK
LYN
CSK
BLK
-log(P)
Log2(Fold Change)

Supplementary Figure 6: Proteomics analysis reveals high selectivity for both covalent and non-covalent BTK PROTACs.

Ramos cells were incubated with each PROTAC or DMSO in quadruplicates for 24h at the designated concentrations, and were then subjected to label-free quantitative proteomics analysis. Each graph plots the Log_2 fold-change of proteins in the treated samples compared to the DMSO controls (X-axis) vs. the -log(p-value) of that comparison in a student's T-test (Y-axis).

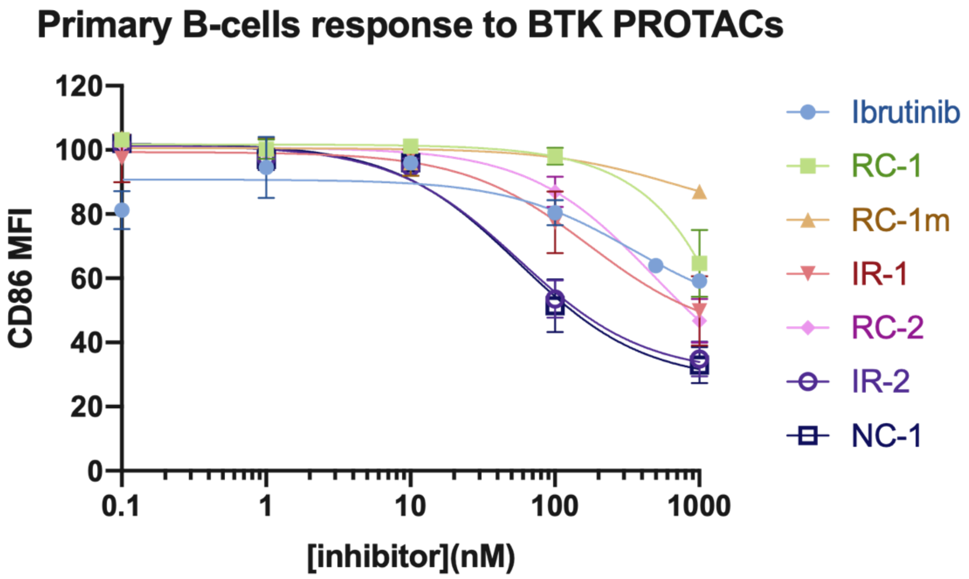

Supplementary Figure 7: PROTACs inhibit B cell receptor signaling more potently than Ibrutinib.

Dose response curves for B cell response after anti-IgM induced activation and treatment with BTK PROTACs or Ibrutinib for 24 hours. The Y-axis shows normalized CD86 Mean fluorescence intensity, where 100% activation is cells stimulated with anti-IgM, and 0% activation is unstimulated cells.

Chemistry Methods:

Materials: were purchased from the following vendors

- BTK inhibitor (free amine), CAS 1022150-12-4: purchased from BLD pharmatech
- Deuterated solvents: Cambridge isotope laboratories
- Non-modified polyethylene glycol: Broadpharm
- Polyethylene glycol (azide-NHS ester): BiochemPEG
- Solvents: Sigma Aldrich
- Other reagents – Sigma Aldrich

Chemistry outline:

The synthesis of the compounds used in this study is outlined in scheme 1.

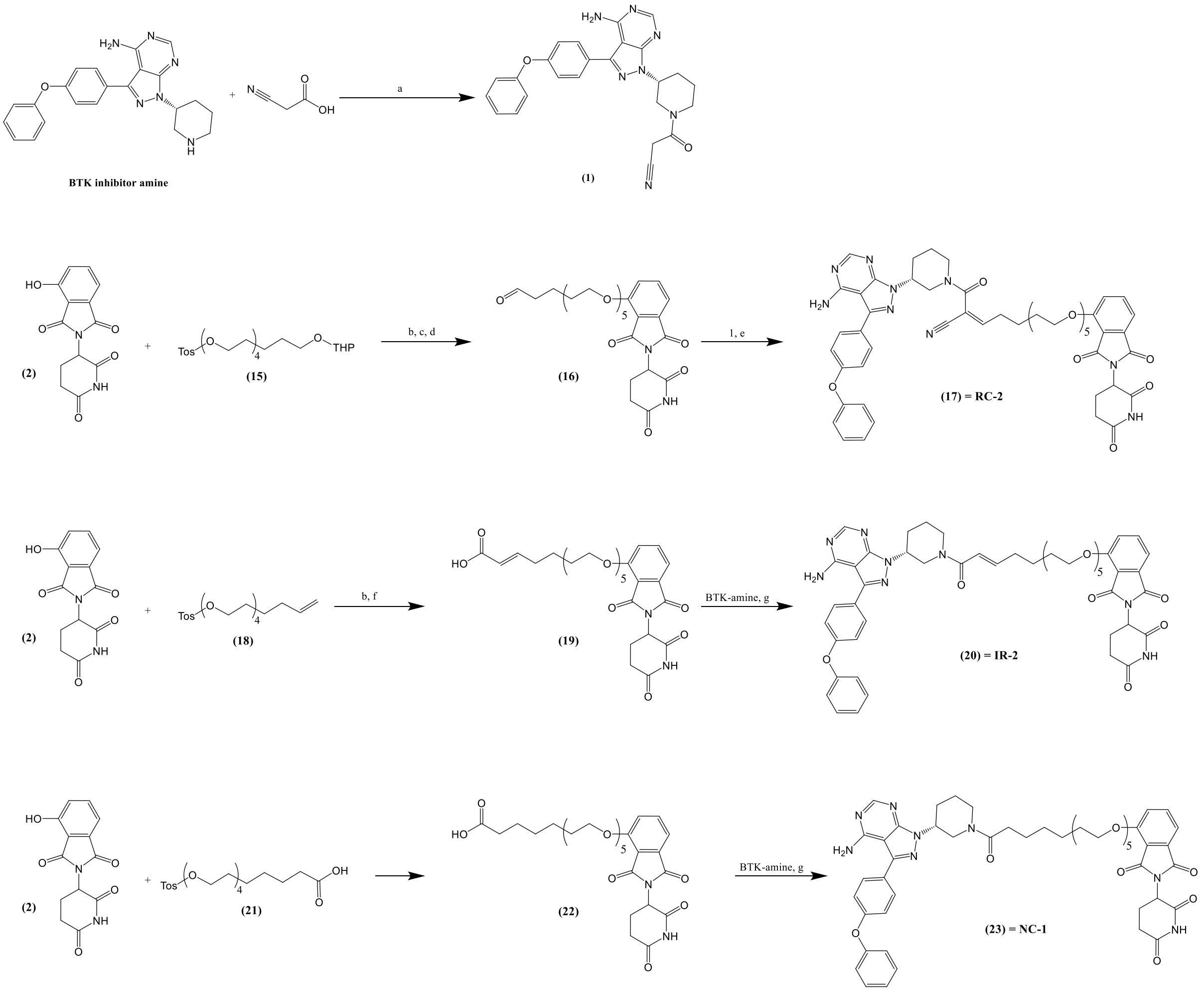

Scheme 1: synthetic scheme: (a) cyanoacetic acid/EDC/DIEA, DCM, RT (b) Na_2CO_3 / DMF, 100°C (c) 5% TFA/methanol, RT (d) Dess Martin Periodinane / DCM, 50C (e) Na_2CO_3 / pyridine, 50°C (f) acrylic acid + Grubbs 2nd gen./THF, 50°C (g) EDC/DIEA/Hobt in DCM, RT

(R)-3-(3-(4-amino-3-(4-phenoxyphenyl)-1H-pyrazolo[3,4-d]pyrimidin-1-yl)piperidin-1-yl)-3-oxopropanenitrile:

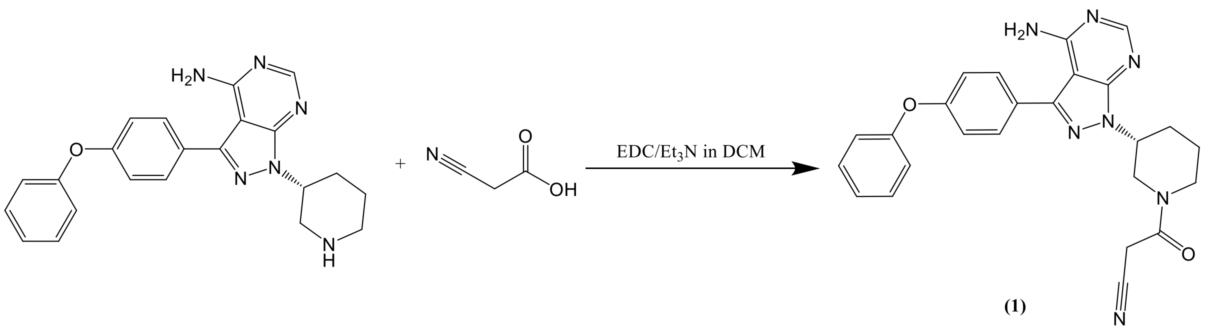

Cyanoacetic acid (1.94 mmol, 165 mg), was dissolved in 10 mL dry dichloromethane. The reaction was cooled on ice and 3.2 mmol EDC hydrochloride (614 mg) and 7.7 mmol of triethylamine (1.07 mL) was added. After 10 minutes 1.29 mmol BTK inhibitor free amine (498 mg) was added and the reaction was warmed to room temperature. After 2 hours the reaction was washed twice with 1M HCl, followed by two washes with 5% $NaHCO_3$, drying over sodium sulfate and evaporating. This yielded **1** as a light brown low density powder, 510 mg (yield 95%).

HR-MS (m/z): Calculated: 453.19; Found: 454.1998 $[M+H]^+$, 476.1815 $[M+Na]^+$.
^{1}H NMR (500 MHz, Acetonitrile-d_3) δ 10.95 (br s, 1H), 8.35 (s, 1H, minor rotamer set A), 8.33 (s, 1H, major rotamer set A), 7.69 – 7.65 (m, 2H), 7.46 (dd, J = 8.6, 7.3 Hz, 1H), 7.25 – 7.20 (m, 2H), 7.15 (d, J = 7.3 Hz, 1H), 6.73 (br s, 1H), 4.99 (tt, J = 9.0, 4.5 Hz, 1H, minor rotamer set B), 4.87 (tt, J = 10.5, 4.3 Hz, 1H, major rotamer set B), 4.59 – 4.53 (m, 1H, major rotamer set C), 4.11 (dt, J = 13.3, 4.5 Hz, 1H, minor rotamer set C), 3.91 – 3.60 (m, 4H), 3.35 – 3.15 (m, 2H), 2.37 – 2.18 (m, 4H, major rotamer set D), 2.03 – 1.87 (m, 4H, minor rotamer set D), 1.85 – 1.74 (m, 1H, major rotamer set E), 1.69 (m, 1H, minor rotamer set E).

^{13}C NMR (126 MHz, Acetonitrile-d_3) δ 162.3, 161.6, 161.5, 158.8, 158.8, 156.4, 153.7, 153.6, 151.8, 146.7, 146.4, 130.1, 130.1, 130.1, 126.2, 126.1, 124.1, 124.1, 119.5, 119.5, 119.3, 117.3, 115.2, 97.3, 53.2, 52.7, 49.2, 45.9, 45.8, 42.0, 29.5, 29.2, 25.1, 25.0, 23.9, 22.6.

Approximated ratio of amide rotamers from 1H-NMR integral ratios: 63:37

2-(2,6-dioxopiperidin-3-yl)-4-hydroxyisoindoline-1,3-dione:

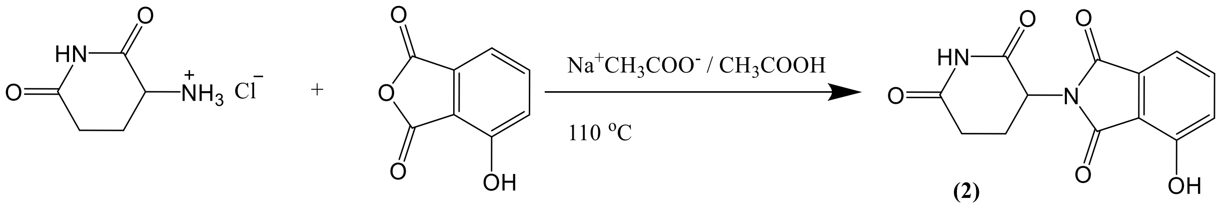

1 g of 2,6-dioxopiperidin-3-aminium (6.1 mmol) and 1 g of 4-hydroxyisobenzofuran-1,3-dione (1 eq.) were dissolved in acetic acid with 2 equivalents of sodium acetate (1 g) and refluxed (110°C) for 12 hours. The solvent was evaporated under reduced pressure, and the residue was washed with ice-cold water and lyophilized. The product was obtained as a brown powder, 1.51 g (90% yield).

HR-MS (m/z): Calculated: 274.06; Found: 297.0492 $[M+Na]^+$.

^{1}H NMR (500 MHz, DMSO-d_6) δ 11.18 (s, 1H), 11.09 (s, 1H), 7.66 (t, J = 7.8 Hz, 1H), 7.33 (d, J = 7.1 Hz, 1H), 7.26 (d, J = 8.4 Hz, 1H), 5.08 (dd, J = 12.8, 5.4 Hz, 1H), 2.89 (ddd, J = 17.0, 13.8, 5.4 Hz, 1H), 2.66 – 2.42 (m, 2H, overlaps with residual DMSO), 2.06 – 1.98 (m, 1H).

^{13}C NMR (126 MHz, DMSO-d_6) δ 173.3, 170.5, 167.5, 166.3, 155.9, 136.9, 133.6, 124.0, 114.8, 114.8, 49.1, 40.5, 40.3, 40.2, 40.0, 39.8, 39.7, 39.5, 31.4, 22.5.

17-hydroxy-3,6,9,12,15-pentaoxaheptadecyl 4-methylbenzenesulfonate:

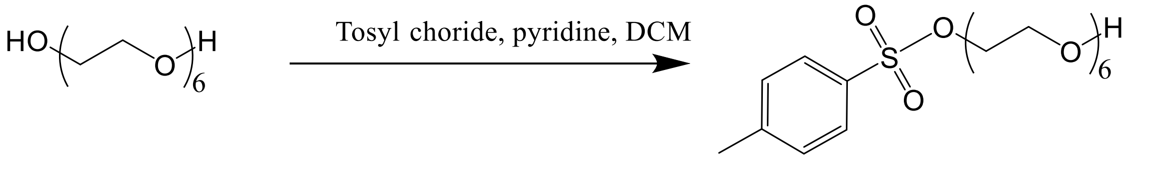

(3)

Hexaethyleneglycol (25 g, 90 mmol, 1.0 equiv.) was dissolved in DCM (150 mL), followed by the addition of pyridine (3.62 mL, 45 mmol, 0.5 equiv.). To the stirred solution was added tosyl chloride (8.58 g, 45 mmol, 0.5 equiv.) in four batches, each 15 minutes apart. After the last addition, the mixture was stirred for 18 h. The reaction mixture was thoroughly freed from

DCM using a rotary evaporator. The resulting residue was treated with 0.1 M HCl (150 mL). The mixture was extracted twice with hexane (50 mL each) to remove excess tosyl chloride. The aqueous layer was washed with three portions of diethyl ether (20 mL each) to remove the ditosylated byproduct, and the extracts were checked by TLC to avoid premature extraction of the monotosylate. The aqueous layer was extracted with three portions of DCM (50 mL each). The combined organic layers were washed with 0.1 M HCl (50 mL), dried over MgSO4, filtered and freed from solvent by rotary evaporation to give the product as a colourless oil (13.2 g weighed mass, 28.71 mmol based on 95% purity by LC/MS, 32%). Monotosylated PEGs of other length were synthesized using analogous procedures.

HR-MS (m/z): Calculated: 436.18; Found: 437.1845 $[M+H]^+$, 459.1674 $[M+Na]^+$

^{1}H NMR (500 MHz, Chloroform-d) δ 7.81 (d, J = 8.0 Hz, 2H), 7.35 (d, J = 7.9 Hz, 2H), 4.17 (t, J = 4.8 Hz, 2H), 3.77 – 3.58 (m, 22H), 3.37 (br s, 1H), 2.46 (s, 3H).

^{13}C NMR (126 MHz, Chloroform-d) δ 144.8, 133.0, 129.8, 128.0, 72.5, 70.7, 70.6, 70.6, 70.5, 70.5, 70.3, 69.3, 68.7, 61.7, 21.7.

Differences in relaxation times between Aryl-H and Alkyl-H lead to incongruent integration results between these two signal sets.

17-((2-(2,6-dioxopiperidin-3-yl)-1,3-dioxoisoindolin-4-yl)oxy)-3,6,9,12,15-pentaoxaheptadecanal:

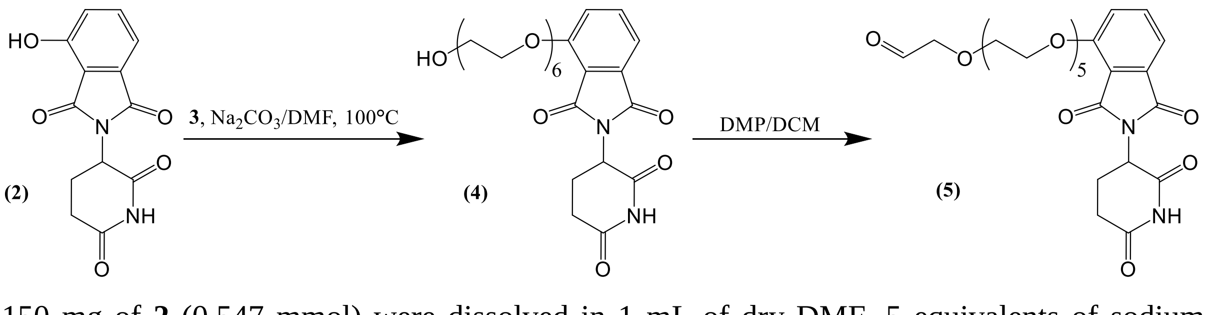

150 mg of **2** (0.547 mmol) were dissolved in 1 mL of dry DMF. 5 equivalents of sodium carbonate (290 mg) were added, and the solution was heated to 100°C under argon. 1.5 equivalents of **3** (358 mg) were dissolved in 250 µl dry DMF and added dropwise to the hot solution while stirring. The reaction was stirred for 3.5 hours under argon. The reaction was

stopped with the addition of 1 mL acetic acid, and the solvent was evaporated under reduced pressure at 75°C. The residue was dissolved in 20%:80% acetonitrile:water (v/v) + 0.1% trifluoroacetic acid and purified by reverse phase HPLC, yielding 212 mg of **4** (71% yield, HR-MS (m/z): Calculated: 538.22; Found: 539.2241 $[M+H]^+$, 561.2076 $[M+Na]^+$). Then, 90 mg of **4** (0.167 mmol) were dissolved in 2 mL DCM and stirred on ice. 2.2 equivalents of Dess Martin Periodinane (156 mg) were added and the reaction stirred on ice for 15 minutes, followed by 2 hours at room temperature. The resulting solution was filtered and evaporated, and the aldehyde **5** was separated from unoxidized alcohol and further oxidized species by HPLC, yielding 23.7 mg of **5** (26% yield) as a highly viscous pale oil (HR-MS (m/z): Calculated: 536.20; Found: 537.2076 $[M+H]^+$, 559.1913 $[M+Na]^+$, 577.2012 $[M+Na+H_2O]^+$).

(E)-18-((R)-3-(4-amino-3-(4-phenoxyphenyl)-1H-pyrazolo[3,4-d]pyrimidin-1-yl)piperidine-1-carbonyl)-1-((2-(2,6-dioxopiperidin-3-yl)-1,3-dioxoisoindolin-4-yl)oxy)-3,6,9,12,15-pentaoxanonadec-17-ene-19-nitrile:

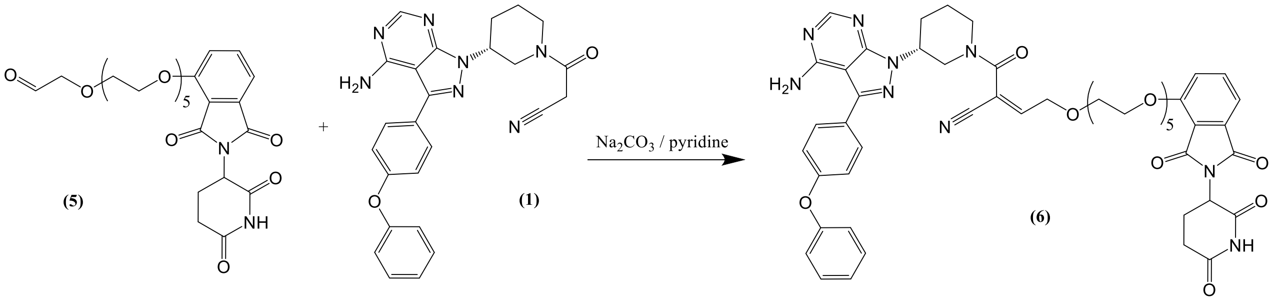

22 mg of 1 (0.0486 mmol) were dissolved in 10 mL of pyridine, and 2.4 mg of sodium carbonate (0.0226 mmol) were added under argon. The aldehyde 5 (30.3 mg, 0.057 mmol) was dissolved in pyridine and added to the reaction, which was stirred at 50°C for 3.5 hours. Dilute reaction conditions and limited reaction times were essential to avoid reaction of condensation products with 1 to give higher molecular weight adducts. The reaction was stopped by addition of 25 mg citric acid and evaporation of the solvent, followed by purification using HPLC. Obtained **6** as a white powder, 12 mg (25% yield).

HR-MS (m/z): Calculated: 972.3814; Found: 972.3887 $[M+H]^+$, 97-94.3701 $[M+Na]^+$

^{1}H NMR (500 MHz, DMSO-d_6) δ 11.11 (s, 1H, NH), 8.40 – 8.27 (m, 1H Ibrutinib-amine-aryl-H), 7.80 (td, *J* = 7.9, 2.6 Hz, 1H, Thalidomide-aryl-H), 7.67 (d, *J* = 8.1 Hz, 2H), 7.52 (dd, *J* = 8.6, 5.1 Hz, 1H, Thalidomide-aryl-H), 7.45 (m, 3H), 7.25 – 7.07 (m, 5H, Ph-O-Ibrutinib amine), 6.86 – 6.24 (m, 1H, Alkenyl-CH), 5.25 – 1.38 (complicated aliphatic region, 36H). Mixture of E/Z diastereomers and amide bond rotamers. Heteroaryl-NH_2 could not be detected.

^{13}C NMR (126 MHz, DMSO-d_6) δ 173.2, 170.4, 167.3, 165.7, 164.1, 158.8, 158.5, 157.8, 156.7, 156.3, 153.6, 150.2, 137.4, 133.7, 130.6, 127.7, 124.3, 120.5, 119.5, 119.4, 116.8, 115.9, 97.6, 70.6, 70.3, 70.3, 70.2, 70.2, 70.2, 69.3, 69.1, 52.7, 49.2, 46.5, 46.0, 31.4, 29.6, 24.3, 22.5. Not all ^{13}C signals could be detected and/or overlapped due to the presence of a hexaethyleneglycol chain.

3,6,9,12,15-pentaoxaoctadec-17-en-1-yl 4-methylbenzenesulfonate:

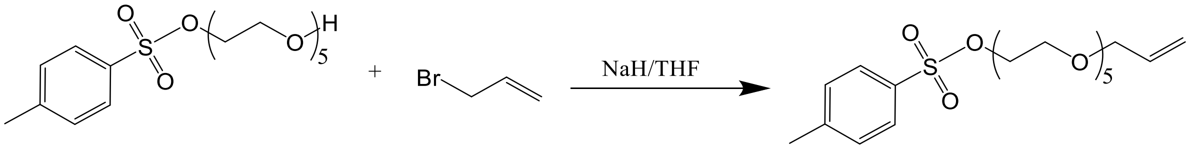

(7)

500 mg of Tos-PEG5-OH (1.29 mmol) was dissolved in dry THF under argon. 2.5 equivalents of allyl bromide were added. The reaction was cooled on ice, and 1.5 equivalents of sodium hydride were added. The solution turned cloudy due to precipitation of sodium bromide. After 20 the reaction was heated to room temperature under argon for 1 hour. TLC using 3% methanol in chloroform confirmed reaction was complete. The reaction was quenched with 1 M HCl, the THF was evaporated in vacuo, and the product was extracted 3 times with dichloromethane, washed with 5% NaHCO3, dried over sodium sulfate, and purified using silica gel chromatography using a gradient of 0 to 10% methanol in chloroform. Obtained the product as a bright yellow oil, 140 mg (yield 25%).

HR-MS (m/z): Calculated: 432.18; Found: 433.1888 $[M+H]^+$, 455.1718 $[M+Na]^+$.

^{1}H NMR (500 MHz, Chloroform-d) δ 7.81 (d, J = 8.2 Hz, 2H), 7.35 (d, J = 8.0 Hz, 2H), 5.92 (ddt, J = 17.4, 10.4, 5.7 Hz, 1H), 5.28 (dd, J = 17.2, 1.6 Hz, 1H), 5.18 (dd, J = 10.4, 1.5 Hz, 1H), 4.19 – 4.15 (m, 2H), 4.03 (dt, J = 5.7, 1.5 Hz, 2H), 3.72 – 3.57 (m, 18H), 2.46 (s, 3H).

^{13}C NMR (126 MHz, Chloroform-d) δ 144.8, 134.8, 133.0, 129.8, 128.0, 117.1, 107.6, 106.4, 77.3, 72.2, 70.7, 70.7, 70.6, 70.6, 70.6, 70.5, 70.5, 69.4, 69.2, 68.7, 67.7, 67.4, 29.5, 29.5, 23.8, 23.8, 21.7.

Differences in relaxation times between Aryl-H, Alkenyl-H and Alkyl-H lead to incongruent integration results between these three signal sets.

(E)-1-((2-(2,6-dioxopiperidin-3-yl)-1,3-dioxoisoindolin-4-yl)oxy)-3,6,9,12,15-pentaoxanonadec-17-en-19-oic acid :

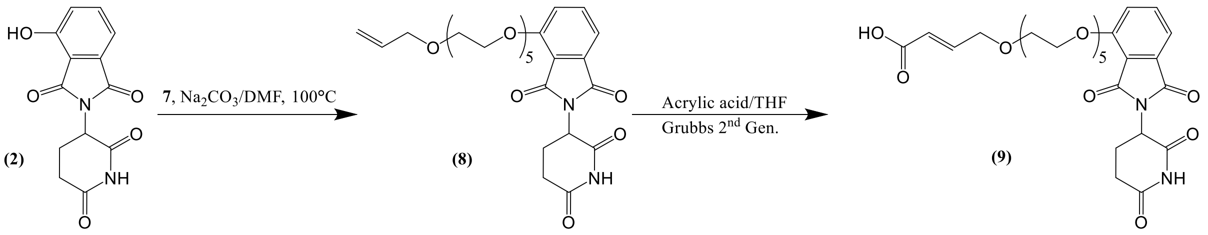

37.5 mg of **2** (0.137 mmol) were dissolved in dry DMF under argon. 5 equivalents of sodium carbonate (69 mg) were added and the solution was heated to 100°C. The tosylate **7** (1.2 equivalents, 71 mg) was dissolved in DMF and added dropwise to the solution. The reaction proceeded for 2 hours under argon, neutralized with 0.5 mL of acetic acid, evaporated and purified by HPLC to yield **8** as a pale, viscous oil (51 mg, 70% yield, HR-MS (m/z): Calculated: 534.32; Found: 535.2280 $[M+H]^+$, 557.2117 $[M+Na]^+$). Then 24 mg of **8** (0.045 mmol) was dissolved in dry THF, and 30 equivalents of acrylic acid (92.5 µl) were added. 0.05 equivalents of Grubbs catalyst 2nd generation was added, and the reaction proceeded under argon at 40°C. The solvent was evaporated and the acid 9 was purified by HPLC as a white powder (10.8 mg, 42 % yield).

HR-MS (m/z): Calculated: 578.21; Found: 601.2010 $[M+Na]^+$.

^{1}H NMR (500 MHz, DMSO-d$_6$) δ 12.32 (s, 1H), 11.10 (s, 1H), 7.82 (dd, J = 8.5, 7.2 Hz, 1H), 7.54 (d, J = 8.5 Hz, 1H), 7.46 (d, J = 7.2 Hz, 1H), 6.81 (dt, J = 15.7, 4.2 Hz, 1H, major isomer set A), 6.33 (dt, J= 11.7, 5.0 Hz, 1H, minor isomer set A), 5.93 (dt, J = 15.7, 2.0 Hz, 1H, major isomer set B), 5.77 (dt, J= 11.7, 2.3 Hz, 1H, minor isomer set B), 5.09 (dd, J = 12.8, 5.4 Hz, 1H), 4.39 – 4.32 (m, 2H), 4.17 – 4.11 (m, 2H), 3.84 – 3.78 (m, 2H), 3.67 – 3.62 (m, 2H), 3.56 – 3.50 (m, 14H), 2.94 – 2.84 (m, 1H), 2.65 – 2.52 (m, 2H), 2.07 – 2.00 (m, 1H).

^{13}C NMR (126 MHz, DMSO-d$_6$) δ 173.24, 170.39, 167.31, 167.27, 165.72, 156.31, 145.27, 137.44, 133.71, 121.57, 120.51, 116.79, 115.85, 70.63, 70.31, 70.29, 70.24, 70.21, 70.18, 70.07, 69.32, 69.30, 69.15, 49.22, 31.42, 22.47.

(Mixture of isomers).

4-(((E)-19-((R)-3-(4-amino-3-(4-phenoxyphenyl)-1H-pyrazolo[3,4-d]pyrimidin-1-yl)piperidin-1-yl)-19-oxo-3,6,9,12,15-pentaoxanonadec-17-en-1-yl)oxy)-2-(2,6-dioxopiperidin-3-yl)isoindoline-1,3-dione:

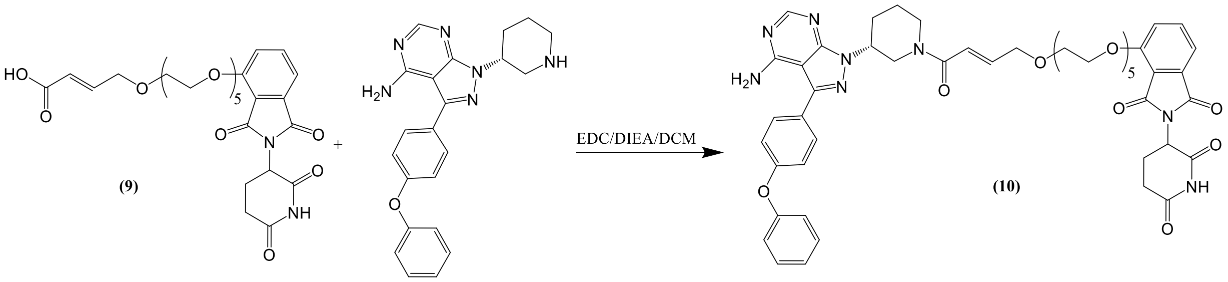

3 mg of **9** (5.19 µmol) was dissolved in dry DCM on ice. 1.5 equivalents of 1-Ethyl-3-(3-dimethylaminopropyl)carbodiimide hydrochloride (1.5 mg) and 3 µl diisopropylethylamine were added. After 5 minutes, 2 equivalents of the BTK inhibitor amine (4 mg) were added, and the reaction proceeded for 1 hour at room temperature. The solvent was evaporated and the product **10** was purified by HPLC as a white powder, 2.4 mg (50% yield).

HR-MS (m/z): Calculated: 946.39; Found: 947.3911 $[M+H]^+$, 969.3732 $[M+Na]^+$.

^{1}H NMR (500 MHz, DMSO-d_6) δ 11.11 (s, 1H), 8.45 (s, 1H), 7.80 (t, J = 7.9 Hz, 1H), 7.70 – 7.63 (m, 2H), 7.53 (d, J = 8.6 Hz, 1H), 7.48 – 7.41 (m, 3H), 7.24 – 7.19 (m, 1H), 7.19 – 7.10 (m, 4H), 6.68 (s, 1H), 6.61 – 6.42 (m, 1H), 5.09 (dd, J = 12.8, 5.4 Hz, 1H), 4.84 – 4.64 (m, 1H), 4.59 – 4.48 (m, 1H), 4.40 – 4.28 (m, 2H), 4.19 – 3.96 (m, 4H), 3.85 – 3.73 (m, 3H), 3.69 – 3.60 (m, 3H), 3.58 – 3.38 (m, 16H), 2.94 – 2.82 (m, 1H), 2.65 – 2.55 (m, 1H), 2.31 – 2.19 (m, 1H), 2.19 – 2.09 (m, 1H), 2.07 – 1.88 (m, 2H), 1.67 – 1.52 (m, 1H).

^{13}C NMR (126 MHz, DMSO-d_6) δ 173.23, 170.38, 167.26, 165.71, 164.79, 158.69, 157.94, 156.62, 156.29, 142.11, 141.62, 137.42, 133.69, 130.62, 124.36, 120.85, 120.49, 119.52, 119.45, 117.48, 116.77, 115.84, 115.15, 97.54, 70.62, 70.29, 70.22, 70.18, 69.93, 69.78, 69.68, 69.32, 69.15, 53.41, 52.86, 49.74, 49.21, 46.16, 45.63, 42.09, 31.41, 29.98, 29.54, 25.24, 23.49, 22.46 (2 unaccounted for carbon peaks indicating some impurities)

2-((tert-butoxycarbonyl)amino)ethyl 4-methylbenzenesulfonate:

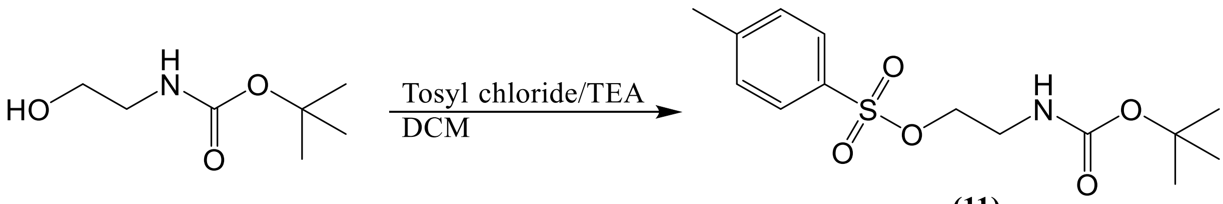

(11)

1 g of Boc-ethanolamine (6.2 mmol) was dissolved in dry DCM on ice, under argon. 2.4 g of tosyl chloride (12.4 mmol) and 4.3 mL of triethylamine (31 mmol) were added, and the reaction proceeded at room temperature for 3 hours. The reaction was quenched with ice, extracted three times with DCM, washed with 1N HCl, 5% sodium bicarbonate and water, dried over sodium sulfate and dried. The residue was purified using flash chromatography using a gradient of 0-10% methanol in chloroform, yielding a waxy solid, 1 g (50%).

^{1}H NMR (500 MHz, Chloroform-d) δ 7.81 (d, J = 8.3 Hz, 2H), 7.37 (d, J = 8.0 Hz, 2H), 4.08 (t, J = 5.1 Hz, 2H), 3.45 – 3.32 (m, 2H), 2.47 (s, 3H), 1.42 (s, 9H).

^{13}C NMR (126 MHz, Chloroform-d) δ 145.0, 132.7, 123.0, 127.9, 79.8, 77.2, 69.5, 39.8, 28.3, 21.7.

2-((2-(2,6-dioxopiperidin-3-yl)-1,3-dioxoisoindolin-4-yl)oxy)ethan-1-aminium:

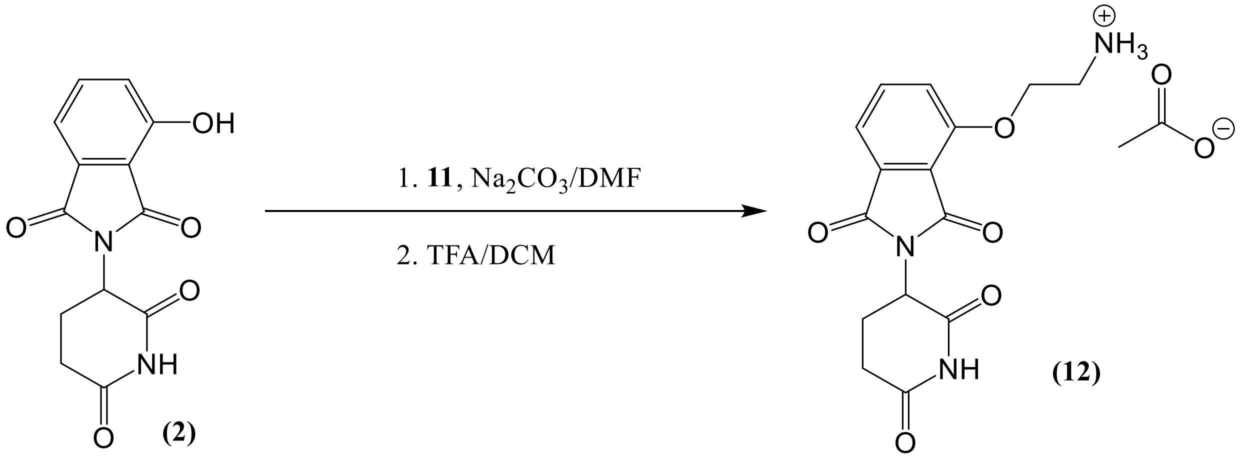

(2) **(12)**

2 was reacted with **11** similar to the procedure for **3** and **8**. The product was HPLC purified, deprotected with 25% trifluoroacetic acid in DCM for 1 hour, and air-dried. The residue was

lyophilized from 40%:40%:20% acetonitrile:water:acetic acid to yield the acetate salt, yield 50%.

^{1}H NMR (500 MHz, DMSO-d_6) δ 11.12 (br s, 1H), 8.05 (br s, 3H), 7.87 (dd, J = 8.5, 7.3 Hz, 1H), 7.56 (dd, J = 16.1, 7.9 Hz, 2H), 5.11 (dd, J = 12.8, 5.4 Hz, 1H), 4.45 (t, J = 5.2 Hz, 2H), 3.28 (d, J = 10.8 Hz, 2H), 2.90 (ddd, J = 17.0, 13.9, 5.4 Hz, 1H), 2.65 – 2.53 (m, 2H), 2.04 (dtd, J = 13.1, 5.4, 2.3 Hz, 1H).

^{13}C NMR (126 MHz, DMSO-d_6) δ 173.24, 170.34, 167.16, 165.70, 155.50, 137.58, 133.73, 121.18, 118.50, 117.54, 116.72, 66.58, 49.27, 38.60, 31.40, 22.49, 21.51.

(contains residual water).

(R,Z)-2-(3-(4-amino-3-(4-phenoxyphenyl)-1H-pyrazolo[3,4-d]pyrimidin-1-yl)piperidine-1-carbonyl)hept-2-en-6-ynenitrile:

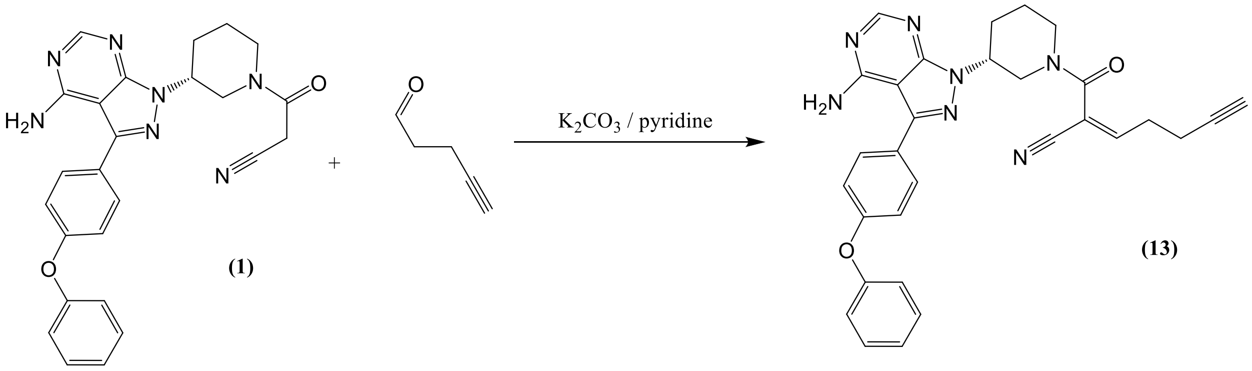

40 mg BTK cyanoacetate were reacted with 30 mg 4-pentynal (4 eq) and 0.5 equivalents potassium carbonate in 4 mL pyridine at 50 C. after 6 hours acetic acid was added, the solvent was evaporated and the product was purified by HPLC as a white powder, 15 mg (33%).

Description of assembly of PROTAC series using combined click and amidation reactions:

Stock solutions were prepared in DMSO as follows: **12**, 200 mM; VHL inhibitor hydrochloride (Molport), 200 mM; bifunctional PEG azide/NHS esters, 200 mM; Triethylamine, 0.5 M; **13**, 200 mM; sodium ascorbate, 200 mM in water, dissolved fresh; TBTA-copper complex, 50 mM

in 1:1 DMSO water (prepared by mixing equal volumes of 100 mM Copper sulfate in water and 100 mM TBTA in DMSO).

1. Reaction 1: 2 µmol of E3 ligase binder with 2 µmol of PEG reagents with 2.5 equivalents of triethylamine in DMSO (10 µl of each stock solution). Reactions were typically complete within 1 hour and monitored by LCMS.

2. Reaction 2: Mixed entire amidation reaction (30 µl of 67 mM) with 2.2 µmol **13** (11 µl), add 4 µmol of sodium ascorbate (20 µl stock), 30 µl water, 100 µl tertbutanol, and 0.6 µl TBTA:copper complex (12 µl stock). Reaction was complete after 2 hours at room temperature.

Products were purified by HPLC, yielding 1-1.9 mg, 50-80% yield.

2-((5-bromopentyl)oxy)tetrahydro-2H-pyran:

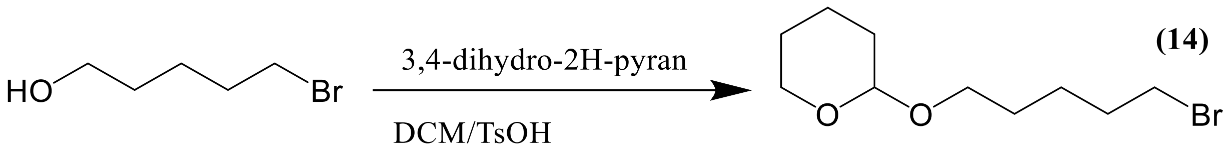

(14)

1 g of 5-bromopentanol (6.15 mmol) was dissolved in 20 mL dry DCM and cooled to 0C on ice. 53 mg of Toluenesulfonic acid hydrate was added, followed by dropwise addition of 0.78 mL 3,4-dihydro-2H-pyran (1.5 equivalents). Reaction proceeded on ice for 12 hours, stopped by addition of aqueous sodium bicarbonate, extracted with DCM, dried over sodium sulfate and evaporated. The residue was purified by flash chromatography hexane:diethylether 30:1. The product was obtained as a clear viscous liquid, 978 mg (63% yield).

^{1}H NMR (500 MHz, Chloroform-d) δ 4.59 (dd, J = 4.4, 2.7 Hz, 1H), 3.88 (ddd, J = 11.0, 7.6, 3.2 Hz, 1H), 3.77 (dt, J = 9.7, 6.6 Hz, 1H), 3.55 – 3.50 (m, 1H), 3.47 – 3.38 (m, 3H), 1.92 (p, J = 7.0 Hz, 2H), 1.88 – 1.81 (m, 1H), 1.76 – 1.71 (m, 1H), 1.69 – 1.50 (m, 8H).

^{13}C NMR (126 MHz, Chloroform-d) δ 98.9, 67.2, 62.4, 33.8, 32.6, 30.8, 28.9, 25.5, 25.0, 19.7.

17-((tetrahydro-2H-pyran-2-yl)oxy)-3,6,9,12-tetraoxaheptadecyl 4-methylbenzenesulfonate:

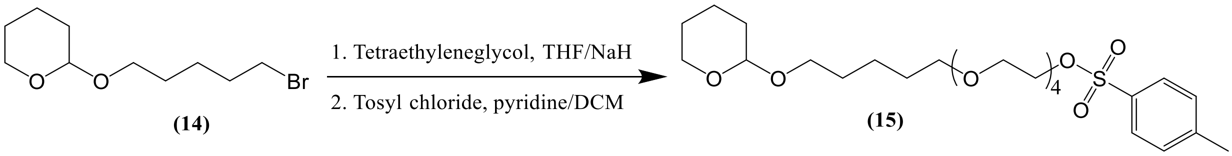

8.13 mmol tetraethylene glycol (1.58 g) were dissolved in dry THF and added slowly at room temperature to a stirring suspension of sodium hydride (327 mg slurry, 8.13 mmol). After 15 minutes at room temperature the reaction was heated to reflux and a solution of 611 mg of **14** (2.42 mmol) in THF was added dropwise. The reflux was continued to reflux for 2.5 hours. The reaction was cooled to room temperature, water was added and the THF was evaporated. The product was extracted with DCM, washed with brine, dried over sodium sulfate and evaporated, yielding 620 mg of crude product (dark yellow oil), which was used in the following step without purification. The product was dissolved in dry DCM on ice and 3 equivalents of pyridine (412 µl) were added. 1.5 equivalents of Tosyl chloride (487 mg) were added in portions while stirring, and the reaction was warmed to room temperature and proceeded for 12 hours. The solvents was then evaporated, the residue was dispersed in a solution of 5% sodium bicarbonate in water, extracted with DCM, washed with brine and dried over sodium sulfate. The product was purified using flash chromatography using 0-10% methanol in chloroform, yielding **15** as a yellow oil, 405 mg (32%). Care was taken to avoid an acidic environment during the reactions and workups to avoid deprotection of the THP group.

^{1}H NMR (500 MHz, Chloroform-d) δ 7.81 (d, J = 8.2 Hz, 2H), 7.36 (d, J = 8.0 Hz, 2H), 4.58 (dd, J = 4.5, 2.7 Hz, 1H), 4.26 – 4.09 (m, 2H), 3.88 (ddd, J = 11.0, 7.6, 3.2 Hz, 1H), 3.78 – 3.32 (m, 18H), 2.46 (s, 3H), 1.90 – 1.33 (m, 13H).

^{13}C NMR (126 MHz, Chloroform-d) δ 144.8, 133.0, 129.8, 127.9, 98.9, 71.4, 70.8, 70.6, 70.6, 70.6, 70.5, 70.1, 69.2, 68.7, 67.5, 62.4, 30.8, 29.6, 29.5, 25.5, 22.8, 21.7, 19.7.

1-((2-(2,6-dioxopiperidin-3-yl)-1,3-dioxoisoindolin-4-yl)oxy)-3,6,9,12-tetraoxaheptadecan-17-al:

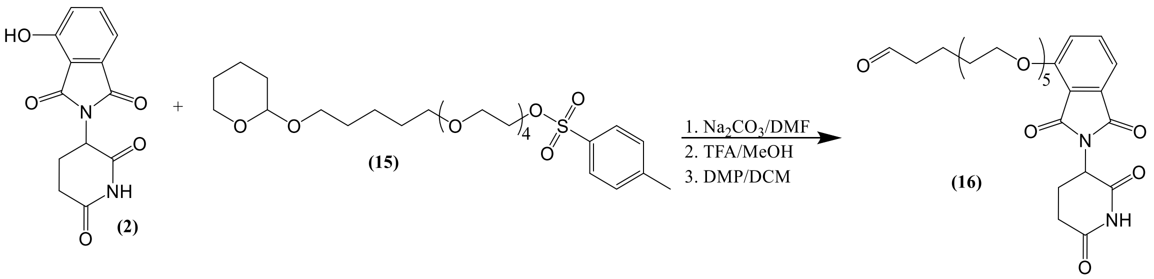

124 mg of 15 (0.24 mmol) was reacted with 2 using the same conditions was described for 4 and 8. Following evaporation of the DMF, the residue was dissolved in methanol + 5% trifluoroacetic acid and stirred at room temperature. Full removal of the THP protecting group (as determined by LC-MS) was achieved within 1.5 hours. The solvent was evaporated and the free alcohol was purified using HPLC as a pale, viscous oil, 85 mg (66% yield). 72 mg of the alcohol (0.134 mmol) was dissolved in DCM and oxidized with 0.16 mmol of dess martin periodinane (68 mg) at 50°C for 2 hours. The solvent was evaporated and the residue was dissolved in 20%:80% acetonitrile:water + 0.1% TFA, filterd and purified using HPLC, yielding 53.6 mg of the purified aldehyde **16** (75% yield) as a pale, highly viscous oil.

HR-MS (m/z): Calculated: 534.22; Found: 557.2118 $[M+Na]^+$.

^{1}H NMR (500 MHz, DMSO-d$_6$) δ 11.11 (s, 1H), 9.64 – 9.66 (t, J= 1.42 Hz, 1H), 7.89 – 7.72 (m, 1H), 7.54 (d, J = 8.8 Hz, 1H), 7.46 (d, J = 7.3 Hz, 1H), 5.09 (dd, J = 12.9, 5.4 Hz, 1H), 4.35 (dd, J = 5.7, 3.5 Hz, 2H), 3.81 (dd, J = 5.6, 3.3 Hz, 2H), 3.70 – 3.61 (m, 2H), 3.58 – 3.52 (m, 2H), 3.52 – 3.40 (m, 10H), 3.36 (t, J = 6.1 Hz, 2H), 2.89 (ddd, J = 17.1, 13.8, 5.4 Hz, 1H), 2.66 – 2.52 (m, 2H), 2.09 – 1.96 (m, 1H), 1.61 – 1.38 (m, 4H).

^{13}C NMR (126 MHz, DMSO-d$_6$) δ 203.89, 173.23, 170.38, 167.27, 165.71, 156.30, 137.44, 133.70, 120.51, 115.85, 70.64, 70.35, 70.30, 70.25, 70.21, 69.90, 69.33, 69.15, 49.22, 43.17, 31.42, 29.00, 22.47, 18.87.

(Z)-18-((R)-3-(4-amino-3-(4-phenoxyphenyl)-1H-pyrazolo[3,4-d]pyrimidin-1-yl)piperidine-1-carbonyl)-1-((2-(2,6-dioxopiperidin-3-yl)-1,3-dioxoisoindolin-4-yl)oxy)-3,6,9,12-tetraoxanonadec-17-ene-19-nitrile

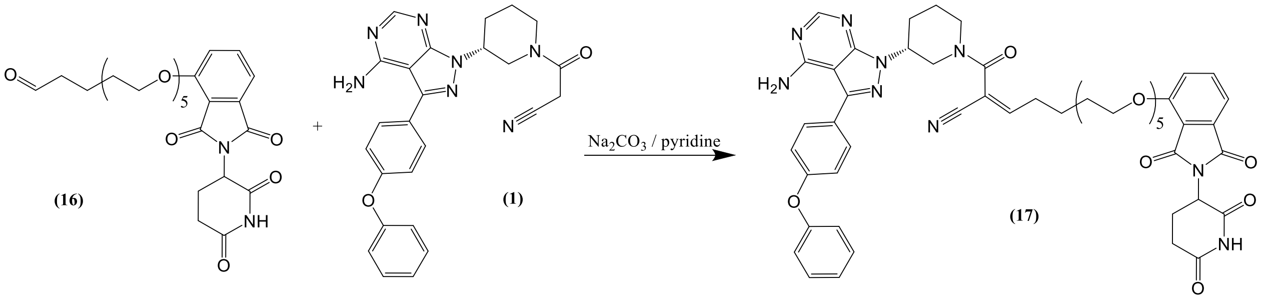

43 mg of **16** (0.08 mmol) were dissolved in 1 mL of pyridine, and 36 mg of **1** (0.08 mmol) were also dissolved in 1 mL of pyridine. 4.24 mg of sodium carbonate (0.04 mmol) were added to the solution of **1**, mixed RT for 5 minutes, and then the aldehyde was added. Reaction proceeded under argon at 50°C for 16 hours. Citric acid was added to neutralize the base and the solvent was evaporated. The product was purified by HPLC, giving **17** as a white powder, 19 mg (25% yield).

HR-MS (m/z): Calculated: 969.40; Found: 970.4078 $[M+H]^+$, 992.3896 $[M+Na]^+$.

^{1}H NMR (500 MHz, DMSO-d_6) δ 11.11 (s, 1H), 8.45 – 8.37 (m, 1H), 7.83 – 7.77 (m, 1H), 7.67 (d, J = 8.5 Hz, 2H), 7.53 (d, J = 8.5 Hz, 1H), 7.45 (t, J = 7.7 Hz, 3H), 7.24 – 7.09 (m, 5H), 7.07 – 6.81 (m, 1H), 5.09 (dd, J = 12.8, 5.4 Hz, 1H), 4.94 – 4.77 (m, 1H), 4.34 (t, J = 4.5 Hz, 2H), 3.80 (t, J = 4.4 Hz, 2H), 3.68 – 3.62 (m, 2H), 3.59 – 3.10 (m, 15H), 2.89 (ddd, J = 17.0, 13.8, 5.4 Hz, 1H), 2.64 – 2.47 (m, 3H), 2.46 – 2.12 (m, 4H), 2.02 (ddd, J = 12.8, 5.4, 2.7 Hz, 2H), 1.77 – 1.60 (m, 1H), 1.56 – 1.35 (m, 4H).

^{13}C NMR (126 MHz, DMSO-d_6) δ 173.2, 170.4, 167.3, 165.7, 162.1, 159.2, 158.9, 158.0, 156.6, 156.3, 153.3, 145.2, 137.4, 133.7, 130.6, 130.6, 127.4, 124.4, 120.5, 119.5, 119.5, 117.5, 116.8, 115.8, 115.2, 115.1, 110.9, 97.6, 70.6, 70.3, 70.2, 70.2, 69.9, 69.3, 69.2, 49.2, 31.5, 31.4, 29.1, 24.5, 22.5.

3*,6,9,12-tetraoxaoctadec-17-en-1-yl 4-methylbenzenesulfonate:*

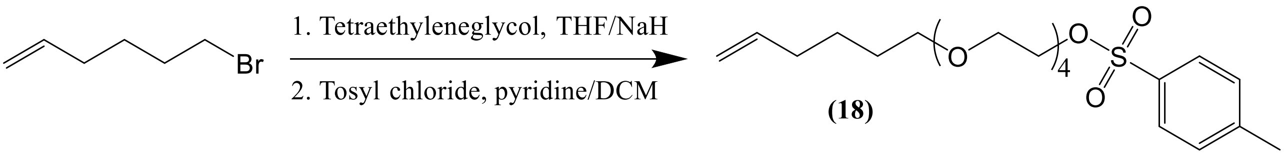

10 mmol PEG4 (1.94 g) was dissolved in dry THF and added dropwise to a suspension of 400 mg NaH/oil dispersion in dry THF at room temperature (400 mg NaH in THF) under nitrogen. After 30 minutes the reaction was heated to reflux and 3 mmol 6-bromo-1-hexene (489 mg) dissolved in THF was added to the solution while it refluxed. After 2 hours the reaction was cooled, neutralized with citric acid and water and the THF was evaporated. The product was extracted 3 times with DCM, washed with brine, dried over sodium sulfate and evaporated, resulting in 550 mg of a brown oil, which was used without purification for the next step. The oil was dissolved in DCM, cooled on ice and 6 mmol pyridine (484 mg) was added. 3 mmol of tosyl chloride (576 mg) was added in portions, and after 30 minutes the reaction was warmed to room temperature and proceeded for 12 hours. The solution was evaporated, and the residue was dispersed in water and extracted with DCM. The organic phase was washed with brine, dried over sodium sulfate, and the crude material was purified using flash chromatography using a gradient of 0-6% methanol in chloroform. The product **18** was obtained as a light brown oil, 400 mg (31% yield).

^{1}H NMR (500 MHz, Chloroform-d) δ 7.82 (d, J = 8.3 Hz, 2H), 7.36 (d, J = 8.0 Hz, 2H), 5.82 (ddt, J = 16.9, 10.2, 6.7 Hz, 1H), 5.02 (dd, J = 17.2, 1.7 Hz, 1H), 4.96 (d, J = 10.3 Hz, 1H), 4.20 – 4.16 (m, 2H), 3.73 – 3.57 (m, 14H), 3.47 (t, J = 6.7 Hz, 2H), 2.47 (s, 3H), 2.11 – 2.04 (m, 2H), 1.65 – 1.57 (m, 2H), 1.50 – 1.40 (m, 2H).

^{13}C NMR (126 MHz, Chloroform-d) δ 144.8, 138.8, 133.0, 129.8, 128.0, 128.0, 114.5, 71.3, 70.8, 70.6, 70.6, 70.5, 70.1, 69.2, 68.7, 33.5, 29.1, 25.4, 21.7.

HR-MS (m/z): Calculated: 430.20; Found: 453.1932 $[M+Na]^{+}$, 469.1673 $[M+K]^{+}$.

(E)-1-((2-(2,6-dioxopiperidin-3-yl)-1,3-dioxoisoindolin-4-yl)oxy)-3,6,9,12-tetraoxanonadec-17-en-19-oic acid:

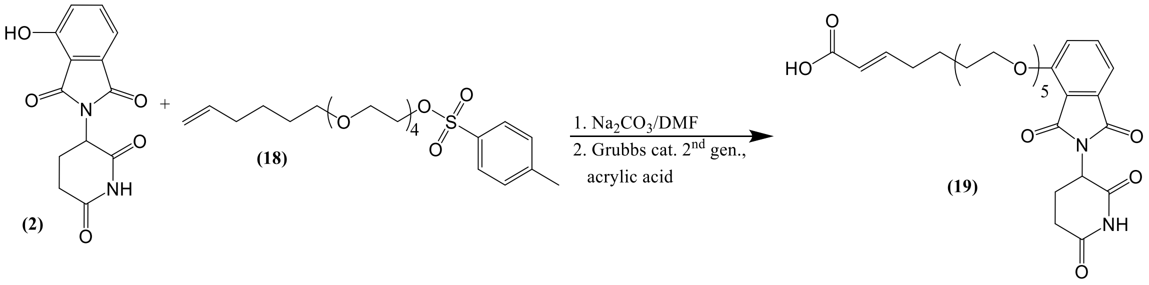

94 mg of **18** (0.219 mmol) was reacted with 50 mg **2** (0.182 mmol) as described before. The product was purified by HPLC. 49.4 mg product (0.092 mmol) was dissolved in 1.5 mL dry THF and mixed with 30 equivalents of acrylic acid (190.4 µl). 0.05 equivalents of Grubbs catalysis 2nd generation (4 mg) was adduct and the reaction was performed at 50°C. The solvent was evaporated and the resulting acid was purified by HPLC as a yellow oil, 46.2 mg (37% yield).

HR-MS (m/z): Calculated: 576.23; Found: 577.2393 $[M+H]^+$, 599.2224 $[M+Na]^+$.

^{1}H NMR (500 MHz, DMSO-d6) δ 11.11 (s, 1H), 7.82 (dd, J = 8.5, 7.2 Hz, 1H), 7.54 (d, J = 8.5 Hz, 1H), 7.46 (d, J = 7.2 Hz, 1H), 6.80 (dt, J = 15.5, 6.9 Hz, 1H), 5.75 (d, J = 15.6 Hz, 1H), 5.09 (dd, J = 12.8, 5.4 Hz, 1H), 4.35 (dd, J = 5.6, 3.5 Hz, 2H), 3.83 – 3.79 (m, 2H), 3.65 (dd, J = 5.8, 3.7 Hz, 2H), 3.63 – 3.58 (m, 4H), 3.54 (dd, J = 5.9, 3.7 Hz, 2H), 3.46 – 3.43 (m, 2H), 3.37 (t, J = 6.1 Hz, 2H), 2.89 (ddd, J = 17.0, 13.8, 5.4 Hz, 1H), 2.63 – 2.56 (m, 1H), 2.19 – 2.15 (m, 2H), 2.06 – 1.98 (m, 1H), 1.81 – 1.72 (m, 5H), 1.53 – 1.38 (m, 4H).

^{13}C NMR (126 MHz, DMSO-d_6) δ 173.2, 170.4, 167.3, 165.7, 156.3, 139.1, 137.4, 133.7, 120.5, 116.8, 115.8, 115.2, 70.6, 70.5, 70.3, 70.3, 70.2, 69.9, 69.3, 69.2, 67.5, 49.2, 33.4, 31.4, 29.1, 25.6, 25.4, 22.5.

4-(((E)-19-((R)-3-(4-amino-3-(4-phenoxyphenyl)-1H-pyrazolo[3,4-d]pyrimidin-1-yl)piperidin-1-yl)-19-oxo-3,6,9,12-tetraoxanonadec-17-en-1-yl)oxy)-2-(2,6-dioxopiperidin-3-yl)isoindoline-1,3-dione:

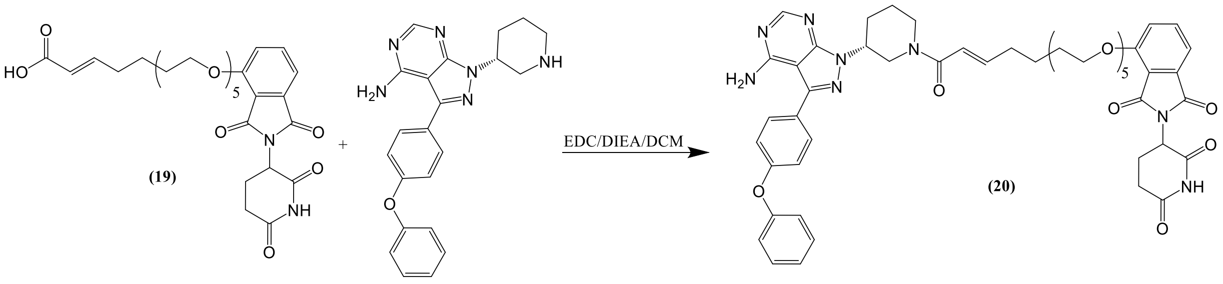

16.5 mg of **19** (0.0286 mmol) were dissolved in DCM and stirred on ice. 1.5 equivalents of EDC (5.5 mg) and 5 equivalents of DIEA (25 µl) were added, and after 10 minutes, 2 equivalents of the BTK inhibitor amine (22 mg) were added. The reaction proceeded at room temperature for 90 minutes, and then the solvent was evaporated and the product **20** was isolated using HPLC as a white powder, 12 mg (44% yield).

HR-MS (m/z): Calculated: 944.41; Found: 945.4130 $[M+H]^+$, 967.3942 $[M+Na]^+$

^{1}H NMR (500 MHz, DMSO-d_6) δ 11.11 (s, 1H), 8.39 (d, J = 6.1 Hz, 1H), 7.80 (t, J = 7.9 Hz, 1H), 7.66 (d, J = 8.0 Hz, 2H), 7.53 (d, J = 8.5 Hz, 1H), 7.45 (t, J = 7.7 Hz, 3H), 7.24 – 7.06 (m, 4H), 6.74 – 6.22 (m, 2H), 5.09 (dd, J = 12.8, 5.4 Hz, 1H), 4.80 – 4.47 (m, 1H), 4.40 – 4.31 (m, 2H), 4.22 – 4.02 (m, 2H), 3.80 (d, J = 4.1 Hz, 2H), 3.64 (d, J = 4.6 Hz, 2H), 3.56 – 2.99 (m, 16H), 2.89 (ddd, J = 16.9, 13.9, 5.4 Hz, 1H), 2.63 – 2.54 (m, 1H), 2.34 – 1.80 (m, 7H), 1.66 – 1.21 (m, 6H).

^{13}C NMR (126 MHz, DMSO-d_6) δ 173.2, 170.4, 167.3, 165.7, 165.2, 157.9, 156.7, 156.3, 146.1, 137.4, 133.7, 130.62, 124.4, 121.1, 120.5, 119.5, 119.5, 116.8, 115.8, 110.9, 97.6, 70.6, 70.4, 70.3, 70.2, 70.2, 69.9, 69.3, 69.2, 49.2, 46.2, 31.8, 31.4, 29.2, 24.9, 22.5. Not all ^{13}C signals could be detected.

1-(tosyloxy)-3,6,9,12-tetraoxanonadecan-19-oic acid:

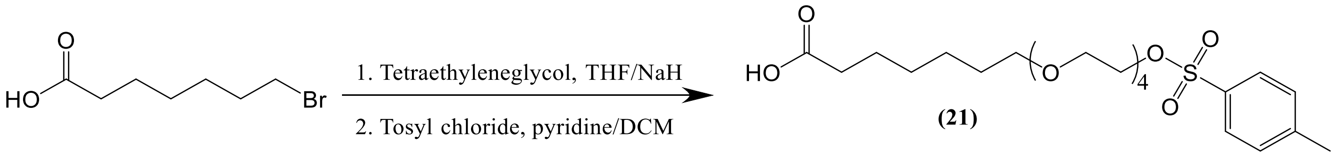

8.13 mmol of tetraethylene glycol (1.58 g) were dissolved in dry THF and added to a slurry of sodium hydride mineral oil dispersion in THF at room temperature (423 mg). After 15 minutes the reaction was warmed to reflux and a solution of 511 mg 7-bromoheptanoic acid (2.42 mmol) in THF was added dropwise, and refluxed for 2 hours. The reaction was stopped by adding water and evaporating the THF. HCl was added to acidify the sample, and the sample was extracted with DCM, washed with brine, dried over sodium sulfate, and evaporated, giving 667 mg of red oil. The product was tosylated as described for **17** and **15**, yielding 47.6 mg of the acid **21** (yield 4%).

HR-MS (m/z): Calculated: 476.21; Found: 499.1986 $[M+Na]^+$, 515.1710 $[M+K]^+$.

^{1}H NMR (500 MHz, Chloroform-d) δ 7.82 (d, J= 8.2 Hz, 2H), 7.36 (d, J = 8.0 Hz, 2H), 3.71 (t, J = 4.9 Hz, 2H), 3.69 – 3.62 (m, 8H), 3.62 – 3.57 (m, 6H), 3.47 (t, J = 6.5 Hz, 2H), 2.47 (s, 3H), 2.36 (t, J = 7.4 Hz, 2H), 1.68 – 1.58 (m, 4H), 1.41 – 1.36 (m, 4H).

^{13}C NMR (126 MHz, Chloroform-d) δ 177.86, 144.78, 133.01, 129.82, 127.99, 71.25, 70.74, 70.64, 70.61, 70.55, 70.50, 70.10, 69.24, 68.69, 33.68, 29.34, 28.77, 25.70, 24.61, 21.65.

1-((2-(2,6-dioxopiperidin-3-yl)-1,3-dioxoisoindolin-4-yl)oxy)-3,6,9,12-tetraoxanonadecan-19-oic acid:

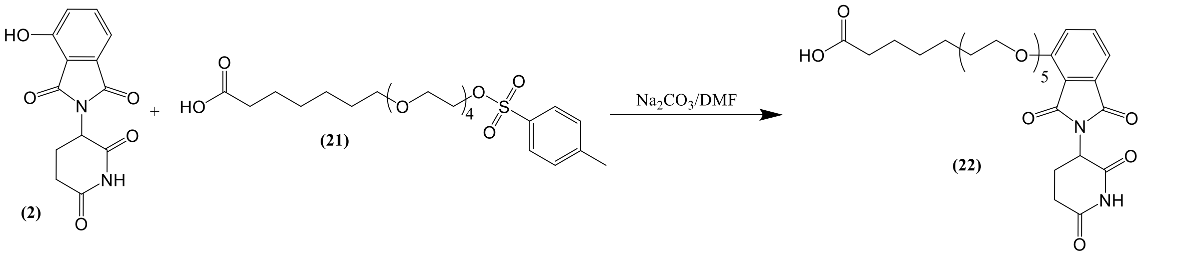

36 mg of **2** (0.131 mmol) as dissolved in dry DMF and 70 mg of sodium carbonate was added. The reaction was warmed to 100°C under nitrogen and a solution of 2 mg **21** (0.088 mmol) in DMF was added dropwise. The reaction continued for 2 hours, and the product was purified by HPLC, yielding 9 mg of **22** as a yellow oil (18% yield).

HR-MS (m/z): Calculated: 578.25; Found: 579.2552 $[M+H]^+$, 601.2380 $[M+Na]^+$.

4-((19-((R)-3-(4-amino-3-(4-phenoxyphenyl)-1H-pyrazolo[3,4-d]pyrimidin-1-yl)piperidin-1-yl)-19-oxo-3,6,9,12-tetraoxanonadecyl)oxy)-2-(2,6-dioxopiperidin-3-yl)isoindoline-1,3-dione

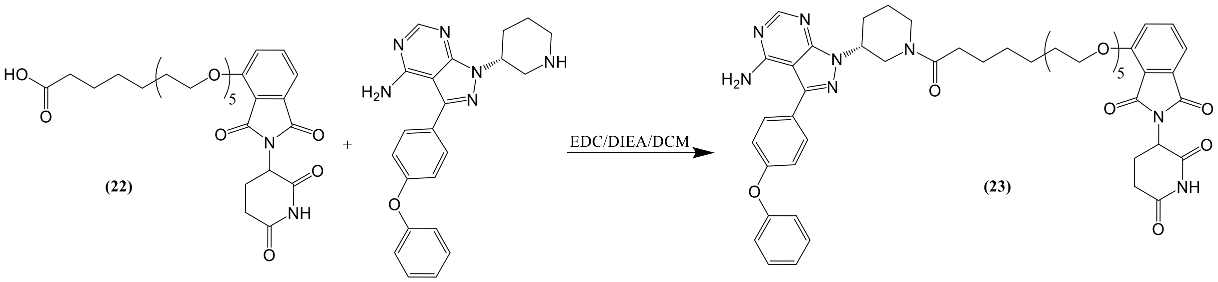

4.5 mg of **22** (7.78 µmol) was dissolved in DCM and cooled on ice. 2 equivalents of EDC (3 mg), 2 equivalents of hydroxybenzotriazole (2.1 mg) and 4.5 equivalents of DIEA (4.7 µl) were added, and the reaction proceeded for 10 minutes on ice. Then 3 equivalents of the BTK inhibitor amine (7.5 mg) were added, and the reaction proceeded for 5 hours at room temperature. The product **23** was purified by HPLC and obtained as a white powder, 6.5 mg (88% yield).

HR-MS (m/z): Calculated: 946.42; Found: 947.4299 $[M+H]^+$, 969.4115 $[M+Na]^+$.

^{1}H NMR (500 MHz, DMSO-d_6) δ 11.11 (s, 1H), 8.38 (d, J = 3.7 Hz, 1H), 7.80 (t, 1H), 7.67 (dd, J = 8.5, 3.6 Hz, 2H), 7.53 (dd, J = 8.7, 3.6 Hz, 1H), 7.49 – 7.42 (m, 3H), 7.20 (t, J = 7.4 Hz, 1H), 7.19 – 7.15 (m, 2H), 7.15 – 7.10 (m, 2H), 5.09 (dd, J = 12.7, 5.3 Hz, 1H), 4.81 - 4.72 (m, 1H, minor rotamer set A), 4.70 – 4.62 (m, 1H, major rotamer set A), 4.51 (dd, J = 12.7, 4.2 Hz, 1H, major rotamer set B), 4.38 – 4.29 (m, 2H), 4.19 (d, J = 12.9 Hz, 1H, minor rotamer set C), 4.03 (dd, J= 13.1, 3.8 Hz, 1H, minor rotamer set B), 3.87 (d, J = 13.6 Hz, 1H, major rotamer set C), 3.84 – 3.75 (m, 2H), 3.70 – 3.61 (m, 2H), 3.56 – 3.52 (m, 2H), 3.52 – 3.40 (m, 8H), 3.38 – 3.29 (m, 2H), 3.18 – 3.09 (m, 1H), 2.94 – 2.83 (m, 2H), 2.66 – 2.53 (m, 2H), 2.40 – 2.08 (m, 4H), 2.07 – 1.98 (m, 1H), 1.97 – 1.84 (m, 1H), 1.68 – 1.58 (m, 1H, major rotamer set D), 1.56 – 1.36 (m, 5H), 1.33 – 1.16 (m, 5H).

^{13}C NMR (126 MHz, DMSO-d_6) δ 173.23, 171.33, 171.14, 170.37, 167.26, 165.71, 158.83, 158.55, 157.91, 157.86, 156.64, 156.30, 153.60, 153.24, 144.96, 144.62, 137.43, 133.70, 130.62, 130.55, 127.68, 127.55, 124.36, 120.50, 119.51, 119.45, 117.52, 116.78, 115.84, 115.19, 97.65, 97.52, 70.73, 70.63, 70.30, 70.24, 70.22, 69.93, 69.91, 69.33, 69.15, 53.44, 52.88, 49.69, 49.21, 45.71, 45.42, 41.48, 32.78, 31.42, 29.98, 29.75, 29.60, 29.56, 29.10, 25.99, 25.91, 25.28, 25.03, 23.88, 22.47.

methyl 5-bromo-2,2-dimethylpentanoate:

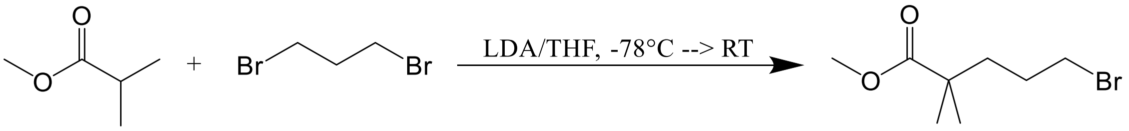

(24)

25 mL of 2M lithium diisopropyl amide in THF/heptane (Sigma) was added to a flask at -78°C under nitrogen. 5.5 mL (5 g, 0.05 mol) of methyl isobutyrate was added dropwise while stirring. The reaction was stirred at -78°C for an hour, and then 0.051 mol of 1,3 dibromopropane (4.2-4.3 mL) was also added dropwise. After 1 hour of stirring at -78°C, the reaction was allowed to warm gradually to room temperature and then kept at room temperature for about 2 hours.

The reaction was poured over a solution of 5% NH_4Cl to quench it, and extracted with ethyl acetate three times. The organic fraction was washed once with 1 N HCl and once with brine, followed by drying on sodium sulfate and evaporating. The product was purified using silica gel chromatorgraphy using a gradient of hexane to ethyl acetate, yielding **24** as a bright pink liquid at ~80% purity based on TLC, yield 3.55 g (~32%).

The product was impure by NMR analysis, but comparison to a spectra of the ethyl ester analogue reported in the literature confirmed the presence of the desired product (Mueller et al, J. Med. Chem., 2004, 47, 6082-6099).

1H NMR (300 MHz, Chloroform-d) δ 3.68 (s, 3H), 3.39 (t, J = 6.5 Hz, 2H), 1.89 – 1.73 (m, 2H), 1.72 – 1.61 (m, 2H), 1.21 (s, 3H).

13C NMR (75 MHz, Chloroform-d) δ 178.0, 51.8, 41.9, 39.2, 33.8, 28.5, 25.2.

5-bromo-2,2-dimethylpentan-1-ol:

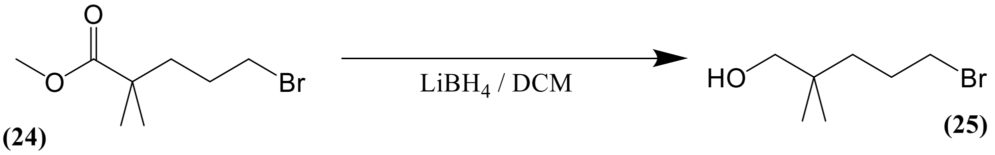

(24) **(25)**

3.4 grams of **24** (0.0152 mol) were reduced as follows. 0.6 g of lithium borohydride (0.0275 mol) were dispersed in dry DCM under nitrogen. 0.9 mL of dry methanol was added while the reaction was stirring inside a room temperature water bath to avoid excessive heating. The ester was then dissolved in DCM and added dropwise. The reaction was stirred under nitrogen overnight at 33C. The reaction was cooled on ice and quenched with cold saturated ammonium chloride solution for 1 hour with shaking. The phases were separated and the product extracted 3 times with 50 mL DCM, followed by two washes with 50 mL ammonium chloride (saturated) and 1 brine wash. The sample was dried over sodium sulfate, filtered through cotton and evaporated, giving 2.8 g of the alcohol **25** as a light pink liquid (0.0143 mol, 94%). **25** was used at the next step without purification.

The product was impure by NMR analysis.

1H NMR (300 MHz, Chloroform-d) δ 3.42 (t, J = 6.9 Hz, 2H), 3.35 (s, 2H), 1.99 – 1.75 (m, 2H), 1.46 – 1.30 (m, 2H), 0.91 (s, 6H).

13C NMR (75 MHz, Chloroform-d) δ 71.7, 37.1, 35.8, 34.7, 27.7, 23.8.

2-((5-bromo-2,2-dimethylpentyl)oxy)tetrahydro-2H-pyran:

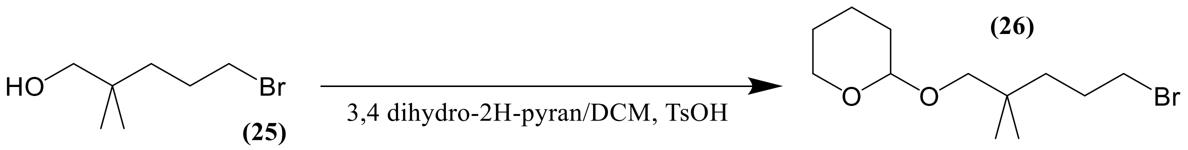

2.3 g of **25** (11.8 mmol) was dissolved in 30 mL dry DCM and cooled to 0C on ice under nitrogen. 0.05 equivalents (112 mg) of Toluenesulfonic acid hydrate was added, followed by dropwise addition of 2.15 mL 3,4-dihydro-2H-pyran (2 equivalents). Reaction proceeded on ice for 4-5 hours until the ice melted, stopped by addition of aqueous sodium bicarbonate, extracted with DCM, dried over sodium sulfate and evaporated. The sample was purified by flash chromatography using 2.5% diethyl ether in 97.5% hexane, yielding **26** as a colorless oil, yield 2.1 g (64 %).

HR-MS (m/z): Calculated: 278.0881; Found: 301.0773 $[M+Na]^+$.

1H NMR (300 MHz, Chloroform-d) δ 4.56 (t, J = 3.3 Hz, 1H), 3.96 – 3.77 (m, 1H), 3.59 – 3.46 (m, 2H), 3.41 (t, J = 6.9 Hz, 2H), 3.01 (d, J = 9.2 Hz, 1H), 1.99 – 1.34 (m, 10H), 0.93 (s, 3H), 0.92 (s, 3H).

13C NMR (75 MHz, Chloroform-d) δ 99.1, 76.2, 62.0, 37.9, 34.8, 34.1, 30.6, 27.9, 25.6, 24.6, 24.5, 19.4.

16,16-dimethyl-17-((tetrahydro-2H-pyran-2-yl)oxy)-3,6,9,12-tetraoxaheptadecyl 4-methylbenzenesulfonate

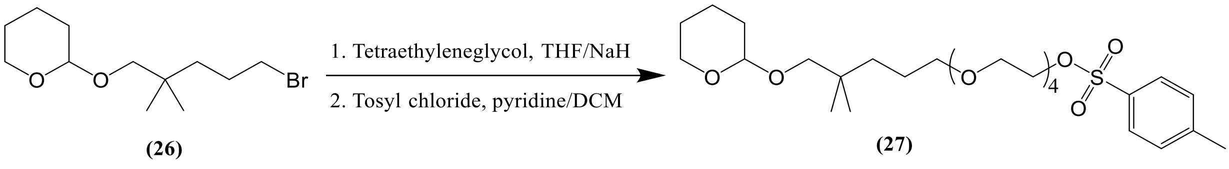

8.13 mmol tetraethylene glycol (1.58 g), was dissolved in dry THF and added slowly to a slurry of 327 mg NaH in dry THF. The reaction was heated to reflux. Then 675 mg of **26** (2.42 mmol) were dissolved in dry THF and added dropwise to the solution under nitrogen. After 4 hours water was added and the THF was evaporated. Citric acid was added slowly until the pH was neutral (but not acidic to avoid THP removal). The sample was extracted with DCM, washed with Brine, filtered over sodium sulfate and evaporated. The sample was then dissolved in dry DCM, 3.5 equivalents of pyridine were added and the sample was cooled on ice. 1.5 equivalents

of tosyl chloride were added in portions, and after 10 minutes on ice the reaction was warmed to room temperature and incubated overnight. The reaction was evaporated and dispersed in saturated sodium bicarbonate, extracted with DCM, washed with brine with added sodium bicarbonate to prevent acidification, filtered over sodium sulfate and purified by flash chromatography, yielding **27** as a viscous yellow oil, 221 mg (17% yield).

HR-MS (m/z): Calculated: 546.2863; Found: 569.2766 $[M+Na]^+$.

1H NMR (400 MHz, Chloroform-d) δ 7.90 (d, J = 8.3 Hz, 2H), 7.44 (d, J = 8.0 Hz, 2H), 4.64 (t, J = 3.5 Hz, 1H), 4.37 – 4.18 (m, 2H), 3.98 – 3.88 (m, 1H), 3.79 (dd, J = 5.6, 4.1 Hz, 2H), 3.75 – 3.71 (m, 6H), 3.68 (s, 5H), 3.62 – 3.48 (m, 4H), 3.09 (d, J = 9.1 Hz, 1H), 2.54 (s, 3H), 1.99 – 1.86 (m, 1H), 1.83 – 1.74 (m, 2H), 1.73 – 1.55 (m, 6H), 1.42 – 1.33 (m, 2H), 1.00 (s, 3H), 0.98 (s, 3H).

13C NMR (101 MHz, Chloroform-d) δ 144.8, 133.0, 129.8, 128.0, 99.2, 77.2, 76.5, 72.4, 70.8, 70.6, 70.6, 70.5, 70.0, 69.2, 68.7, 62.0, 35.3, 34.1, 30.7, 25.6, 24.6, 24.4, 24.3, 21.7, 19.5.

1-((2-(2,6-dioxopiperidin-3-yl)-1,3-dioxoisoindolin-4-yl)oxy)-16,16-dimethyl-3,6,9,12-tetraoxaheptadecan-17-al

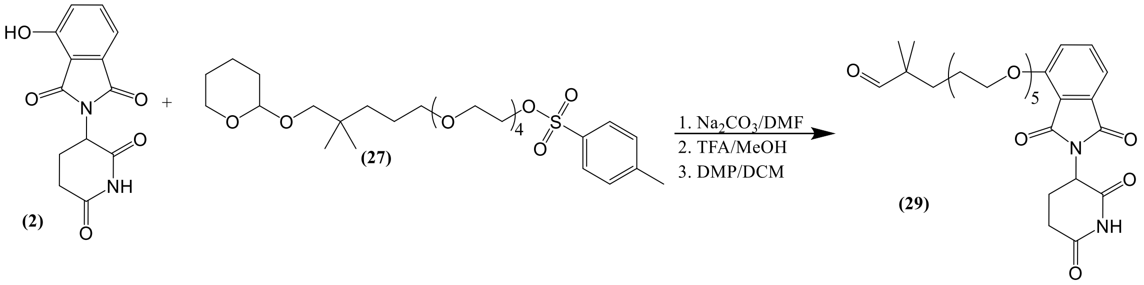

100 mg of **27** (0.183 mmol) were dissolve in 200 µl dry DMF. 1.5 equivalents of **2** (75 mg) were dissolve in 300 µl DMF and 7.5 equivalents sodium carbonate (145 mg) were. Argon was bubbled through the sample and the solution was heated to 100°C followed by addition of the solution of **27**. After 2 hours the DMF was evaporated and the residue was dissolved in 15 mL of 10% TFA in methanol. Deprotection was complete within 1 hour according to LC-MS. The methanol and TFA were evaporated and the thalidomide alcohol conjugate **28** was purified by HPLC as a high viscous pink oil, yield 63 mg (61%).

HR-MS (m/z): Calculated: 564.2683; Found: 565.2750 $[M+H]^+$.

1H NMR (400 MHz, DMSO-d_6) δ 11.18 (s, 1H), 7.95 – 7.87 (m, 1H), 7.62 (d, J = 8.5 Hz, 1H), 7.55 (d, J = 7.2 Hz, 1H), 5.17 (dd, J = 12.8, 5.4 Hz, 1H), 4.54 – 4.34 (m, 2H), 3.89 (dd,

J = 5.6, 3.5 Hz, 2H), 3.76 – 3.70 (m, 2H), 3.66 – 3.34 (m, 13H), 3.15 (s, 2H), 3.03 – 2.92 (m, 1H), 2.72 – 2.62 (m, 1H), 2.15 – 2.06 (m, 1H), 1.54 – 1.46 (m, 2H), 1.37 – 1.30 (m, 1H), 1.28 – 1.18 (m, 2H), 0.85 (s, 6H).

13C NMR (101 MHz, DMSO-d_6) δ 173.2, 170.4, 167.3, 165.7, 156.3, 137.5, 133.7, 120.5, 116.8, 115.9, 71.8, 70.6, 70.3, 70.3, 69.9, 69.3, 69.2, 49.2, 35.2, 35.0, 31.4, 24.5, 24.4, 22.5.

28 was then dissolved in dry DCM and 1.2 equivalents of dess martin periodinane were added (56.4 mg). The reaction proceeded at 45°C for three hours, filtered and evaporated. The residue was purified by HPLC, yielding 29 mg of pure **29** (46 % yield) as a viscous pink oil.

HR-MS (m/z): Calculated: 562.2527; Found: 585.2430 $[M+Na]^+$.

1H NMR (400 MHz, DMSO-d_6) δ 11.10 (s, 1H), 9.40 (s, 1H), 7.81 (dd, J = 8.5, 7.2 Hz, 1H), 7.53 (d, J = 8.5 Hz, 1H), 7.46 (d, J = 7.2 Hz, 1H), 5.08 (dd, J = 12.8, 5.4 Hz, 1H), 4.34 (dd, J = 5.8, 3.5 Hz, 2H), 3.80 (t, J = 4.6 Hz, 2H), 3.64 (dd, J = 5.9, 3.7 Hz, 2H), 3.56 – 3.40 (m, 9H), 3.38 – 3.29 (m, 6H), 2.88 (ddd, J = 17.0, 13.9, 5.4 Hz, 1H), 2.08 – 1.96 (m, 1H), 1.55 – 1.21 (m, 4H), 1.13 (d, J = 3.5 Hz, 1H), 1.09 – 1.04 (m, 1H), 0.96 (s, 2H), 0.82 (dd, J = 10.0, 7.1 Hz, 1H).

13C NMR (101 MHz, DMSO-d_6) δ 207.1, 173.2, 170.4, 167.3, 165.7, 156.3, 137.5, 133.7, 120.5, 116.8, 115.9, 49.2, 45.5, 40.6, 38.5, 35.3, 35.2, 33.5, 31.4, 29.8, 27.8, 27.6, 25.4, 24.6, 24.6, 22.9, 22.5, 21.4.

18-((R)-3-(4-amino-3-(4-phenoxyphenyl)-1H-pyrazolo[3,4-d]pyrimidin-1-yl)piperidine-1-carbonyl)-1-((2-(2,6-dioxopiperidin-3-yl)-1,3-dioxoisoindolin-4-yl)oxy)-16,16-dimethyl-3,6,9,12-tetraoxanonadec-17-ene-19-nitrile

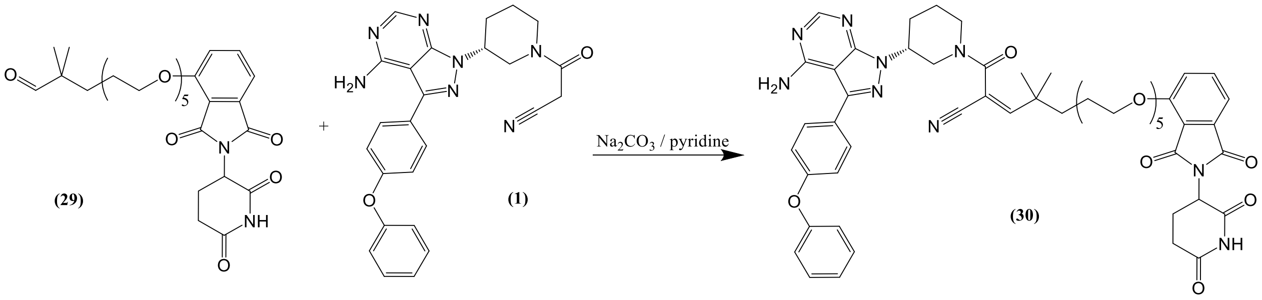

7.27 mg of **29** (0.0129 mmol) and 5.84 mg of **1** (0.0129 mol) were dissolved in pyridine (400 ul) and added to a pressure vessel. 5 equivalents of sodium carbonate (6.8 mg) were added, the vessel was flushed with argon, and the reaction was warmed to 150C. After 50 hours citric acid was added to neutralize the reaction, the pyridine was evaporated, and the residue was purified by HPLC, yielding **30** as a white powder.

Not all expected 13C-NMR resonances could be detected.

HR-MS (m/z): Calculated: 997.4334; Found: 998.4390 $[M+H]^+$.

1H NMR (400 MHz, DMSO-d_6) δ 11.18 (s, 1H), 8.37 (s, 1H), 7.88 (t, J = 7.9 Hz, 1H), 7.74 (d, J = 8.6 Hz, 2H), 7.60 (d, J = 8.6 Hz, 1H), 7.52 (t, J = 8.0 Hz, 2H), 7.34 – 7.15 (m, 5H), 6.91 – 6.71 (m, 1H), 5.50 (t, J = 32.9 Hz, 2H), 5.16 (dd, J = 12.9, 5.4 Hz, 1H), 4.96 (s, 1H), 4.42 (t, J = 4.5 Hz, 2H), 3.87 (t, J = 4.6 Hz, 2H), 3.77 – 3.67 (m, 2H), 3.64 – 3.47 (m, 13H), 2.96 (ddd, J = 17.3, 14.1, 5.4 Hz, 1H), 2.66 (d, J = 17.9 Hz, 1H), 2.44 – 2.30 (m, 1H), 2.25 (d, J = 7.0 Hz, 1H), 2.16 – 2.03 (m, 2H), 1.99 (s, 1H), 1.86 – 1.70 (m, 1H), 1.64 – 1.05 (m, 14H).

13C NMR (101 MHz, DMSO-d_6) δ 173.2, 170.4, 167.3, 165.7, 163.4, 162.9, 160.0, 157.7, 156.7, 156.3, 137.4, 133.7, 130.6, 130.5, 128.1, 124.3, 120.5, 119.5, 119.4, 116.8, 115.8, 108.2, 97.8, 70.6, 70.3, 70.2, 70.2, 69.9, 69.3, 69.2, 49.2, 31.4, 29.5, 26.6, 26.5, 22.5, 21.5.

References

(1) Bradshaw, J. M.; McFarland, J. M.; Paavilainen, V. O.; Bisconte, A.; Tam, D.; Phan, V. T.; Romanov, S.; Finkle, D.; Shu, J.; Patel, V.; Ton, T.; Li, X.; Loughhead, D. G.; Nunn, P. A.; Karr, D. E.; Gerritsen, M. E.; Funk, J. O.; Owens, T. D.; Verner, E.; Brameld, K. A.; Hill, R. J.; Goldstein, D. M.; Taunton, J. Prolonged and Tunable Residence Time Using Reversible Covalent Kinase Inhibitors. Nat. Chem. Biol. 2015, 11 (7), 525–531. https://doi.org/10.1038/nchembio.1817.

(2) Cox, J.; Mann, M. MaxQuant Enables High Peptide Identification Rates, Individualized p.p.b.-Range Mass Accuracies and Proteome-Wide Protein Quantification. Nat. Biotechnol. 2008, 26 (12), 1367–1372. https://doi.org/10.1038/nbt.1511.

(3) Cox, J.; Hein, M. Y.; Luber, C. A.; Paron, I.; Nagaraj, N.; Mann, M. Accurate Proteome-Wide Label-Free Quantification by Delayed Normalization and Maximal Peptide Ratio Extraction, Termed MaxLFQ. Mol. Cell. Proteomics 2014, 13 (9), 2513–2526. https://doi.org/10.1074/mcp.M113.031591.

(4) Sun, Y.; Zhao, X.; Ding, N.; Gao, H.; Wu, Y.; Yang, Y.; Zhao, M.; Hwang, J.; Song, Y.; Liu, W.; Rao, Y. PROTAC-Induced BTK Degradation as a Novel Therapy for Mutated BTK C481S Induced Ibrutinib-Resistant B-Cell Malignancies. Cell Res. 2018, 28 (7), 779–781. https://doi.org/10.1038/s41422-018-0055-1.
